# A meta-analysis of potential biomarkers associated with severity of coronavirus disease 2019 (COVID-19)

**DOI:** 10.1186/s40364-020-00217-0

**Published:** 2020-08-31

**Authors:** Celestin Danwang, Francky Teddy Endomba, Jan René Nkeck, Dominic Leandry Angong Wouna, Annie Robert, Jean Jacques Noubiap

**Affiliations:** 1 Epidemiology and Biostatistics Unit, Institut de Recherche Expérimentale et Clinique, Université catholique de Louvain, Brussels, Belgium; 2 Psychiatry Internship Program, University of Bourgogne, 21000 Dijon, France; 3 Health Economics & Policy Research and Evaluation for Development Results Group, Yaoundé, Cameroon; 4 Department of Internal medicine, Faculty of Medicine and Biomedical Sciences, University of Yaoundé I, Yaoundé, Cameroon; 5 General medical practices unit, Bertoua Regional Hospital, Bertoua, Cameroon; 6 Centre for Heart Rhythm Disorders, University of Adelaide and Royal Adelaide Hospital, Adelaide, Australia

**Keywords:** Prognostic biomarkers, COVID-19, Meta-analysis

## Abstract

**Background:**

Prognostic factors for the Coronavirus disease 2019 (COVID1–9) are not well established. This study aimed to summarize the available data on the association between the severity of COVID-19 and common hematological, inflammatory and biochemical parameters.

**Methods:**

EMBASE, MEDLINE, Web of sciences were searched to identify all published studies providing relevant data. Random-effects meta-analysis was used to pool effect sizes.

**Results:**

The bibliographic search yielded 287 citations, 31 of which were finally retained. Meta-analysis of standardized mean difference (SMD) between severe and non-severe COVID-19 cases showed that CK-MB (SMD = 0.68,95%CI: 0.48;0.87; *P-value:*< 0.001), troponin I (SMD = 0.71, 95%CI:0.42;1.00; *P-value:*< 0.001), D-dimer (SMD = 0.54,95%CI:0.31;0.77; *P-value:*< 0.001), prothrombin time (SMD = 0.48, 95%CI:0.23;0.73; *P-value:* < 0.001), procalcitonin (SMD = 0.72, 95%CI: 0.34;1,11; *P-value:*< 0.001), interleukin-6 (SMD = 0.93, 95%CI: 0.25;1.61;*P-value:* 0.007),C-reactive protein (CRP) (SMD = 1.34, 95%CI:0.83;1.86; *P-value:*< 0.001), ALAT (SMD = 0.53, 95%CI: 0.34;0,71; *P-value:*< 0.001), ASAT (SMD = 0.96, 95%CI: 0.58;1.34; *P-value:* < 0.001), LDH (SMD = 1.36, 95%CI: 0.75;1.98; *P-value:*< 0.001), CK (SMD = 0.48, 95%CI: 0.10;0.87; *P-value:*0.01), total bilirubin (SMD = 0.32, 95%CI: 0.18;0.47;*P-value:* < 0.001), γ-GT (SMD = 1.03, 95%CI: 0.83;1.22; *P-value:* < 0.001), myoglobin (SMD = 1.14, 95%CI: 0.81;1.47; *P-value:*< 0.001), blood urea nitrogen (SMD = 0.32, 95%CI: 0.18;0.47;*P-value:*< 0.001) and Creatininemia (SMD = 0.18, 95%CI: 0.01;0.35; *P-value:*0.04) were significantly more elevated in severe cases, in opposition to lymphocyte count (SMD = -0.57, 95%CI:-0.71; − 0.42; *P-value:* < 0.001) and proportion of lymphocytes (SMD = -0.81, 95%CI: − 1.12; − 0.49; *P-value:*< 0.001) which were found to be significantly lower in severe patients with other biomarker such as thrombocytes (SMD = -0.26, 95%CI: − 0.48; − 0.04; *P-value:*0.02), eosinophils (SMD = − 0.28, 95%CI:-0.50; − 0.06; *P-value:*0.01), haemoglobin (SMD = -0.20, 95%CI: − 0.37,-0.03; *P-value:*0.02), albuminemia (SMD-1.67,95%CI -2.40; − 0.94; *P-value:*< 0.001), which were also lower. Furthermore, severe COVID-19 cases had a higher risk to have lymphopenia (RR =1.66, 95%CI: 1.26;2.20; *P-value*:0.002), thrombocytopenia (RR = 1.86, 95%CI: 1.59;2.17; *P-value*: < 0.001), elevated procalcitonin level (RR = 2.94, 95%CI: 2.09–4.15; *P-value*:< 0.001), CRP (RR =1.41,95%CI: 1.17–1.70; *P-value*:0.003), ASAT(RR =2.27, 95%CI: 1.76;2.94; *P-value*:< 0.001), CK(RR = 2.61, 95%CI: 1.35;5.05; *P-value*: 0.01), Creatininemia (RR = 3.66, 95%CI: 1.53;8.81; *P-value*: 0.02) and LDH blood level (RR = 2.03, 95%CI: 1.42;290; *P-value*: 0.003).

**Conclusion:**

Some inflammatory (procalcitonin, CRP), haematologic (lymphocyte, Thrombocytes), and biochemical (CK-MB, Troponin I, D-dimer, ASAT, ALAT, LDH, γ-GT) biomarkers are significantly associated with severe COVID-19. These biomarkers might help in prognostic risk stratification of patients with COVID-19.

## Introduction

Coronavirus disease 2019 (COVID-19) which emerged in Wuhan, China, has spread to almost all countries and regions of the world, becoming one of the most lethal pandemic after the Spanish flu in 1918–1920. It is caused by an RNA virus (2019 novel coronavirus or 2019-nCoV or SARS-CoV-2). As of July 13, 2020, a total of 12,768,307 confirmed COVID-19 cases and 566,654 related deaths have been reported [1]. Beyond its important morbidity and mortality and the huge burden of health care systems, COVID-19 has a massive societal and economic impact globally.

To date, there is no established curative treatment for COVID-19. Although some drugs such as hydroxychloroquine are integrated in treatment guidelines or under investigation in interventional studies, the management of COVID-19 is mostly supportive. [2–4] Identifying biological abnormalities induced by COVID-19 may contribute to a better understanding of the pathophysiology of the disease and ultimately guide the development of targeted adjuvant therapies besides antivirals drugs. Furthermore, such information on the biological profile of COVID-19 can guide clinicians in the assessment and treatment of these patients.

Clinically, most of COVID-19 cases (80%) are either asymptomatic or have mild forms. [4, 5] However, about 13.8 and 6.1% have severe and critical life-threatening disease that require admission to hospital and sometimes in the intensive care unit. [5] In the context of outstretched heath care systems and limited resources, risk stratification is pivotal to identify patients who the most need in-hospital and intensive management. Biomarkers along with some clinical factors might help to predict adverse outcomes among COVID-19 patients. Hence, we conducted this systematic review and meta-analysis to summarize available data on the association between some common hematological, inflammatory, biochemical parameters and the severity of COVID-19.

## Methods

This review is reported according to the MOOSE (Meta-analysis Of Observational Studies in Epidemiology) guidelines [6].

### Search strategy and selection criteria

We searched PubMed, EMBASE and Web of sciences from inception to April 18, 2020, to identify studies in English or French reporting biological work-up results in patients with biologically confirmed (with polymerase chain reaction assays) COVID-19. The search strategies are presented in the appendix (Supplementary Table 1 and 2). Furthermore, the reference list of eligible studies was analysed to identify potential additional data sources. For duplicates publications or studies conducted on the same group of patients, the study with the largest sample size was considered. We excluded studies from which we could not obtain data on the prevalence of abnormalities or the mean and standard deviation (or median and interquartile range) of the reported biomarker in severe and non-severe patients.

### Data extraction and management

Two investigators (CD and FTE) independently assessed the articles retrieved from the literature search to determine their potential eligibility based on titles and abstracts. The full texts of selected articles were then downloaded and assessed for final inclusion. Discrepancies were resolved by discussion and consensus.

An electronic data abstraction form was used by four investigators (CD, FTE, JRN and DLAW) to extracted relevant information in accordance with the objectives of the review. One investigator (JJN) crosscheck extracted data. For each study data extracted included: surname of the first author, year of publication, country and city where the study was conducted, study design, timing of data collection, sample size, proportion of males, study sample size, biological abnormalities reported, mean and standard deviation (or median and interquartile range) of reported biomarkers.

All patients presenting blood oxygen saturation ≤ 93%; respiratory failure; septic shock; multiple organ dysfunction; dyspnea;respiratory rate greater than 30/min, PaO2/FiO2 ratio < 300, and/or lung infiltrates > 50% of the lung field within 24–48 h were considered as severe cases as recommended by the World Health Organization. [5]

### Data synthesis and analysis

A DerSimonian and Laird random-effects meta-analysis model was used to obtain the pooled effect size within the statistical software R (version 3.6.2). For studies reporting median and interquartile range of the biomarker, only those with a reported number of cases greater than 25 were included in the standardized mean difference (SMD) meta-analysis. Prior to pooling effect sizes, the standard deviation of each mean value was computed from the interquartile range by taking the difference between Q3 and Q1 and dividing by 1.35 as recommended in the Cochrane handbook of systematic review [7]. Pooled OR and SMD of each biomarker are reported with their 95% confidence intervals.

Cochran’s χ^2^ and the I^2^ tests were used respectively to assess the presence and the amount of heterogeneity, with the cut-off of I^2^ values of 25, 50 and 75% representing low, medium and high heterogeneity respectively [8, 9]. Publication bias was assess by the inspection of the Funnel plot and the Egger test (*p* < 0.10) [10]. Unless otherwise specify, a *p* value < 0.05 was considered as statistically significant for all analysis.

## Results

### Characteristics of included studies

The bibliographical search yielded 287 eligible articles, of which 31 were finally retained for the systematic review, and 16 for the meta-analysis as depicted in the study selection process (Supplementary Figure 1). Two studies were conducted in Singapore [11, 12] and all the others in China in 2020 [13–41] (Table 1). Twelve of the included studies were conducted in Wuhan. Twenty-eight studies had collected data retrospectively while three were prospective. All included studies except one (which was cross sectional) were case series with patients having a mean age ranging from 9 to 70.5 years in severe cases and from 7.5 to 59.7 years in non-severe cases. The proportion of males ranged from between 40.7 and 73%. Three studies reported data on survivors and non-survivors (Table 1).

**Table 1:** Characteristics of included studies

Cai	Qi	Case serie	China	Shenzhen	Single center	Hospital	Retrospective	Non-servere Vs Servere	417	85	NR	233	NR	NR	NR
**Cai**	Q	Case serie	China	Shenzhen	Single center	Referal hospital	Retrospective	Non-servere Vs Servere	298	58	62.5	240	41	Liver diseases:13.9%; Type II diabetes:13.8%; Hypertension:37.9%; Cardiac diseases:22.4%;	Liver diseases:8.3%; Type II diabetes:4.2; Hypertension:10.4; Cardiac diseases:5.0%
**Chen**	X	Case serie	China	Chongqing	Single center	Hospital	Retrospective	Non-servere Vs Servere	78	15	NR	63	NR	NR	NR
**Chen**	G	Case serie	China	Wuhan	Single center	Hospital	Retrospective	Moderate Vs Severe	21	11	61	10	52	Hypertension: 36.4%; diabetes: 18.2%	Hypertension: 10%; diabetes:10%
**Fan**	B	Case serie	Singapore	Unclear	Single center	Hospital	Prospective	ICU VS No-ICU	67	9	54	58	41	NR	NR
**Feng**	Y	Case serie	China	Wuhan	Multicenter	Hospital	Retrospective	Moderate Vs Critical	476	70	61	352	51	Hypertension: 35.7%; diabetes 8.6%; cardiovascular diseases: 17.1%; malignancy: 8.6%;COPD: 15.7%;immunosuppression: 7.1%;	Hypertension: 20.7%;diabetes 9.1%; cardiovascular diseases 6%; malignancy: 1.4%;COPD: 2.3%;immunosuppression: 0.6%;
**Guan**	W	Case serie	China	Unclear	Multicenter	Unclear	Retrospective	Admission to an intensive care unit (ICU), use of mechanical ventilation, or death	1099	173	52	926	45	Hepatitis B: 2.4%; Diabetes:16.2%;Hypertention: 23.7%;COPD:3.5%; CHD: 5.8%; Cerebro-Vas diseases: 2.3%;Immunodeficiency:0%;Cancer: 1.7%	Hepatitis B: 0.6%; Diabetes: 5.7%;Hypertention:13.4%;COPD: 0.6%; CHD: 1.8%; Cerebro-Vas diseases:1.2%;Immunodeficiency:0.2%;Cancer:0.8%
**Han**	H	Cross sectional	China	Wuhan	Single center	Hospital	Prospective	Ordinary Vs Severe	94	35	NR	49	NR	NR	NR
**Huang**	C	Case serie	China	Wuhan	Single center	Hospital	Prospective	Non-servere Vs Servere	41	13	49	28	49	Diabetes:8%;Hypertention: 15%;COPD:8%; CVD: 23%;Immunodeficiency:%;Cancer: 0%	Diabetes: 25%;Hypertention: 14%;COPD:0%;CVD: 11%;Cancer: 4%
**Li**	Y	Case serie	China	Huazhong	Single center	Department of Thoracic Surgery	Retrospective	Non-servere Vs Servere	25	9	NR	16	NR	Hypertention: 11.1%;COPD:44.4%;diabetes:11.1%;CHD: 11.1	Hypertention:6.3%;COPD:6.3%;diabetes: 0%;CHD:18.8
**Li**	K	Case serie	China	Unclear	Single center	Hospital	Retrospective	Non-servere Vs Servere	83	25	53.7	58	41	Hypertention:8%;COPD: 16.0%;diabetes:28.0%;CHD: 4.0%	Hypertention5.2%;COPD: 1.7%;diabetes:0.0%;CHD:0.0%
**Liu**	Y	Case serie	China	Shenzhen	Single center	Hospital	Retrospective	Mechanical ventilation VS No Mechanical ventilation	12	6	64.5	6	52	Hypertention:17.6%;COPD:%;diabetes:16.7%;CHD:33.3%;cancer:0%;chronic liver diseases:0%; chronic renal diseases:16.7%	
**Liu**	W	Case serie	China	Wuhan	Multicenter	Hospital	Retrospective	Common Vs Severe	78	8	NR	70	NR	Hypertention:10.3%;COPD: 5.1%;diabetes:6.4%;cancer:5.1%	
**Mao**	L	Case serie	China	Wuhan	Multicenter	Neurology	Retrospective	Non-servere Vs Servere	214	88	58.2	126	48.9	Hypertention:36.4%; diabetes: 17.0%; cardiac/cerebral disease: 8.0%; Cancer: 5.7%; chronic kidney disease:2.3%	Hypertention: 15.1%; diabetes:11.9%; cardiac/cerebral disease:6.3%; Cancer:6.3%; chronic kidney disease:3.2%
**Qian**	G	Case serie	China	Zhejiang	Multicenter	Hospital	Retrospective	Non-servere Vs Servere	91	9	66	82	49	NR	NR
**Qin**	C	Case serie	China	Tongji	Single center	Hospital	Retrospective	Non-servere Vs Servere	452	286	61	166	53	Hypertention:36.7%;COPD: 3.1%;diabetes:18.5%;cancer:3.5%;CVD:8.4;CeVD:2.8%; chronic liver diseases: 1%;Tuberculosis:2.4%; chronic kidney diseases: 2.1%	Hypertention:18.1%;COPD: 1.8%;diabetes:13.3%;cancer:2.4%;CVD:1.8%;CeVD:1.8%; chronic liver diseases:1.8%;Tuberculosis:1.2%; chronic kidney diseases: 2.4%
**Qiu**	H	Case serie	China	Zhejiang	Multicenter	Paediatric	Retrospective	Mild Vs Moderate	36	19	9	17	7.5	NR	NR
**Qu**	R	Case serie	China	Huizhou	Single center	Hospital	Retrospective	Non-servere Vs Servere	30	3	60	27	49.4	NR	NR
**Ruan**	Q	Case serie	China	Jin,Tongji	Multicenter	Hospital	Retrospective	Death Vs Discharge	150	68	NR	82	NR	NR	NR
**Wan**	S	Case serie	China	Chongqing	Single center	Hospital	Retrospective	Non-servere Vs Servere	135	40	56	95	44	Hypertention:10%;COPD:10%;diabetes:22.5%;cancer: 7.5%;CVD:15%;chronic liver diseases:2.5%; pulmonary diseases:2.5%	Hypertention:9.4%;COPD:0%;diabetes: 3.1%;cancer:1.5%;CVD:1%; chronic liver diseases: 1%;Tuberculosis: %;diseases:0%
**Wang**	Z	Case serie	China	Wuhan	Multicenter	Hospital	Retrospective	Non-servere Vs Servere	69	14	70.5	55	37	Hypertention:36%;COPD: 14%;diabetes:43%;cancer: 7%;CVD:36%;chronic liver diseases:0%;	Hypertention: 7%;COPD: 4%;diabetes: 2%;cancer: 5%;CVD:5%;chronic liver diseases:2%
**Wang**	R	Case serie	China	Fuyang	Single center	Hospital	Retrospective	Non-servere Vs Servere	125	25	49.4	100	39.7	Comorbidities: 48%	Comorbidities:22%
**Wang**	D	Case serie	China	Wuhan	Single center	Hospital	Retrospective	Non-servere Vs Servere	138	36	66	102	51	Hypertention:58.3%;COPD: 8.3%;diabetes:22.2%;cancer:11.1%;CVD:25.0%;chronic liver diseases:0%; CeVD:16.7%;HIV:0%	Hypertention:21.6%;COPD:1.0%;diabetes:5.9%;cancer: 5.9%;CVD:10.8%;chronic liver diseases:3.9%; CeVD:1.0%;HIV:2.0%
**Wu**	C	Case serie	China	Wuhan	Single center	Hospital	Retrospective	Survivor Vs Non-survivor	201	44	68.5	40	50	^a^Hypertention:36.4%; diabetes:25.0%; CVD:9.1%	^a^Hypertention:17.5%; diabetes:12.5%;CVD:2.5%
**Xu**	Y	Case serie	China	Unclear	Single center	Radiology	Retrospective	Mild/Moderate/Severe/critical	50	3	NR	9	NR	NR	NR
**Yang**	X	Case serie	China	Wuhan	Single center	Hospital	Retrospective	Survivor Vs Non-survivor	52	32	64.6	20	59.7	^a^COPD:6%;diabetes:22%;cancer:3%;CVD:9%;chronic liver diseases:0%; CeVD:22%	^a^COPD:10%;diabetes:10%;cancer:5%;CVD:10%;chronic liver diseases:0%;CeVD:0%
**Young**	B	Case serie	Singapore	NR	Multicenter	Hospital	Retrospective	Require Vs not require supp Oxygen	18	6	56	12	37	Comorbidities:67%	Comorbidities:8%
**Yun**	L	Case serie	China	Shanghai	Single center	Hospital	Retrospective	No-servere Vs Servere	292	21	65.5	271	48.7	NR	NR
**Zhang**	J	Case serie	China	Wuhan	Multicenter	Hospital	Retrospective	Non-servere Vs Servere	140	58	64	82	51.5	Hypertention:37.9%; COPD:3.4%; diabetes:13.8%;CHD:6.9%;liver diseases:6.9%; CeVD: 3.4%	Hypertention:24.4%; COPD:0%; diabetes:11.0%;CHD:3.7%;liver diseases:5.0%; CeVD: 1.2%
**Zheng**	F	Case serie	China	Changsha	Single center	Hospital	Retrospective	Non-servere Vs Servere	161	30	57	131	40	Hypertention:40%; COPD:6.7%; diabetes:6.7%; CHD:6.7%;chronic liver diseases:3.1%; CeVD:3.3%	Hypertention:7.6%; COPD:3.1%; diabetes:3.8%;CHD:1.5%;chronic liver diseases:0%;CeVD:2.3%
**Zhou**	F	Case serie	China	Wuhan	Multicenter	Pulmonary hospital	Retrospective	Survivor Vs Non-survivor	191	54	69	137	52	^a^ Hypertention:48%; COPD:7%; diabetes: 31%; cancer:0%; CHD:14%;chronic kidney diseases:4%	^a^ Hypertention: 23%; COPD:1%; diabetes: 14%; cancer:1%; CHD:1%;chronic kidney diseases:1%

^a^Survivor Vs Non-survivors; *CeVD* Cerebrovascular diseases; *CVD* Cardiovascular diseases; *COPD* chronic obstructive pulmonary diseases; *CHD* coronary heart diseases; *NR* Not reported

### Full blood count abnormalities

Severe COVID-19 cases had significantly lower lymphocyte count (SMD = -0.57, 95%CI:-0.71; − 0.42; *P-value:* < 0.001; *n* = 3337), proportion of lymphocytes (SMD = -0.81, 95%CI: − 1.12; − 0.49; *P-value:*< 0.001;*n* = 1974), thrombocytes (SMD = -0.26, 95%CI: − 0.48; − 0.04; *P-value:*0.02;*n* = 2064), eosinophils (SMD = − 0.28, 95%CI:-0.50; − 0.06; *P-value:*0.01; *n* = 436) and haemoglobin (SMD = -0.20, 95%CI: − 0.37,-0.03; *P-value:*0.02;*n* = 843). They had a significantly higher neutrophil count (SMD = 0.52, 95%CI: 0.28;0.76; *P-value:*< 0.001; *n* = 2156) (Table 2). Moreover, non-survivor cases of COVID-19 had a significantly lower level of lymphocytes (SMD = -0.67, 95% CI: − 1.18; − 0.17; *P-value:* 0.009; *n* = 327) and higher level of white blood cells (SMD = 0.89, 95%CI:0.04; 1.75; *P-value:* 0.04; *n* = 275) (Table 3).

**Table 2:** Summary results of the meta-analysis of mean values of each Biomarker in severe vs non-severe cases

Anomalies	SMD (95% CI)	P-value	Heterogeneity I2 (%)	Number of studies	Sample size for severe	Sample size for Non-severe
Inflammation						
Procalcitonin	0.72 (0.34;1,11)	< 0.001	87	6	467	1042
CRP	1.34 (0.83;1.86)	< 0.001	95	9	670	1304
IL-6	0.93 (0.25;1.61)	0.007	93	3	369	506
ESR	0.27 (−0.16;0.70)	0.22	90	4	435	1029
Blood routine						
Lymphocytes count	-0.57 (−0.71; −0.42)	< 0.001	61	12	888	2449
Lymphocytes %	-0.81 (−1.12; − 0.49)	< 0.001	62	3	367	306
Thrombocytes	-0.26 (− 0.48; − 0.04)	0.02	72	7	445	1619
Eosinophils	−0.28 (− 0.50; − 0.06)	0.01	0	2	114	322
Neutrophils	0.52 (0.28;0.76)	< 0.001	80	9	646	1510
Haemoglobin	−0.20 (− 0.37; − 0.03)	0.02	0	4	165	678
Monocytes	−0.09(− 0.27;0.08)	0.30	14	4	372	426
White Blood Cells	0.13 (−0.14;0.39)	0.35	90	11	1133	2566
CD3+ T	−0.77(−0.95; − 0.59)	< 0.001	0	2	307	437
Cardiac injury biomarkers						
CK-MB	0.68(0.48;0.87)	< 0.001	30	4	185	965
Troponin I	0.71(0.42;1.00)	< 0.001	0	2	57	373
Biochemestry						
CK	0.48(0.10;0.87)	0.01	89	7	343	1317
Myoglobin	1.14(0.81;1.47)	< 0.001	66	3	149	863
ALAT	0.53(0.34;0,71)	< 0.001	68	10	507	1785
ASAT	0.96(0.58;1.34)	< 0.001	91	9	453	1650
Albumin	−1.67(−2.40; −0.94)	< 0.001	93	4	185	855
Creatinemia	0.18(0.01;0.35)	0.04	49	8	368	1417
Blood urea nitrogen	0.58(0.23;0.93)	0.001	83	5	277	920
Total bilirubin	0.32(0.18;0.47)	< 0.001	28	7	344	1253
LDH	1.36(0.75;1.98)	< 0.001	95	7	343	1317
Potassium	−0.10(−0.43;0.23)	0.55	79	3	248	1061
Sodium	−0.19(− 0.72;0.34)	0.49	91	3	231	983
γ-GT	1.03(0.83;1.22)	< 0.001	0	2	143	473
Blood clothing						
PT	0.48(0.23;0.73)	< 0.001	16	3	111	246
D-dimer	0.54(0.31;0.77)	< 0.001	69	7	348	1235
aPT	0.17(−0.23;0.57)	0.40	74	4	164	274
Fibrinogen	0.09(−0.56;0.74)	0.78	77	2	56	320

*SMD* Standardized mean difference; *CRP* C-reactive protein; *CK* Creatine kinase; *IL-6* interleukin-6; *ALAT* alanine amino-transferase;

*ASAT* aspartate amino-transferase; *LDH* Lactate dehydrogenese; *PT* prothrombin time; *aPT* activated partial thromboplastin;

**Table 3:** Summary results of the meta-analysis of mean values of each Biomarker in non-survivor and survivor cases

Anomalies	SMD (95% CI)	P-value	Heterogeneity I2 (%)	Number of studies	Sample size for Non-survivor	Sample size survivor
Inflammation						
IL-6	1.23(0.77;1.68)	< 0.001	61	2	98	177
Blood routine						
Lymphocytes count	−0.67(−1.18; −0.17)	0.009	75	3	130	197
White Blood Cells	0.89(0.04; 1.75)	0.04	90	2	98	177
Biochemestry						
Creatinemia	−0.09(−0.46;0.27)	0.62	12	2	76	60
Total bilirubin	0.55(0.20;0.89)	0.002	0	2	76	60
LDH	1.48(0.57;2.40)	0.002	90	2	98	170
Ferritin	0.76(−0.33;1.90)	0.18	93	2	90	122
Blood clothing						
PT	0.30(−0.14;0.75)	0.18	68	3	130	188

*SMD* Standardized mean difference; *IL-6* interleukin-6; *LDH* Lactate dehydrogenese; *PT* prothrombin time; *CD3+ T* CD3+ positif T lymphocytes

Furthermore, cases of severe COVID-19 were more likely to present lymphopenia (RR =1.66, 95%CI: 1.26;2.20; *P-value:*0.002; *n* = 1636), and thrombocytopenia (RR =1.86, 95%CI: 1.59;2.17; *P-value:* < 0.001; *n* = 1226) but not leukopenia (RR =0.93,95%CI: 0.46;1.86; *P-value:* 0.81; *n* = 1684) (Table 4). More details on the meta-analysis and forest plots are available in the appendix (Supplementary file).

**Table 4:** Summary results of the meta-analysis of odd ratio of each Biomarker in severe vs non-severe cases

Anomalies	RR (95% CI)	P-value	Heterogeneity I2 (%)	Number of studies	Sample size for severe	Sample size for non-severe
Inflammation						
Eleveted procalcitonin	2.94 (2.09; 4.15)	< 0.001	32	9	321	941
Eleveted CRP	1.41 (1.17; 1.70)	0.003	0	8	328	1021
Blood routine						
Lymphopenia	1.66(1.26;2.20)	0.002	30	11	411	1225
Leucopenia	0.93(0.46;1.86)	0.81	83	10	384	1300
Thrombocytopenia	1.86(1.59;2.17)	< 0.001	0	5	250	976
Thrombocytosis	0.88(0.26;2.95)		0	2	53	122
Biochemestry						
Eleveted LDH	2.03(1.42;2.90)	0.003	57	7	239	899
Eleveted ASAT	2.27(1.76;2.94)	< 0.001	26	8	354	1184
Eleveted ALAT	160(1.34;1.90)	0.002	60	5	283	1042
Eleveted CK	2.61(1.35;5.05)	0.01	37	6	248	841
Eleveted Creatininemia	3.66(1.53; 8.81)	0.02	0	4	227	857
Eleveted total bilirubin	1.42(0.18;11.26)	0.28	0	2	158	725
Blood clothing						
D-dimer	1.50(0.89; 2.56)	0.08	0	3	156	510

*OR* odd ratio; *CRP* C-reactive protein; *CK* Creatine kinase; *IL-6* interleukin-6; *ALAT* alanine amino-transferase;

*ASAT* aspartate amino-transferase; *LDH* Lactate dehydrogenese

### Blood clotting abnormalities

There was a significant difference in D-dimer and prothrombin time (PT) between severe and non-severe cases, with severe cases having a higher D-dimer level (SMD = 0.54,95%CI:0.31;0.77; *P-value:* < 0.001; *n* = 1583) and prothrombin time (SMD = 0.48, 95%CI:0.23;0.73; *P-value:* < 0.001; *n* = 357).

### Increased cardiac injury biomarkers in severe cases

Troponin I blood level were significantly higher in severe patients (SMD = 0.71, 95%CI: 0.42;1.00; *P-value:*< 0.001; *n* = 430) as well as CK-MB levels (SMD = 0.68,95%CI: 0.48;0.87; *P-value:*< 0.001; *n* = 1150), and this was consistent across all studies included in the meta-analysis of the above-mentioned biomarkers (Table 2).

### Inflammation

Severe COVID-19 cases had significantly higher blood level of procalcitonin (SMD = 0.72, 95%CI: 0.34;1,11; *P-value:* < 0.001; *n* = 1509), interleukin-6 (SMD = 0.93, 95%CI: 0.25;1.61; *P-value:* 0.007; *n* = 875),and C-reactive protein (SMD = 1.34, 95%CI:0.83;1.86; *P-value:* < 0.001; *n* = 1974) (Table 2). Moreover, IL-6 was significantly higher in non-survivor compare to survivor cases (SMD = 1.23, 95%CI: 0.77;1.68; *P-value:*< 0.001; *n* = 275).

Severe cases were more likely to have a higher procalcitonin level (RR =2.94, 95%CI: 2.09; 4.15; *P-value:* < 0.001; *n* = 1262), and CRP (RR =1.41,95%CI: 1.17; 1.70; *P-value:* 0.003; *n* = 1349) (Table 4).

### Biochemical abnormalities

Overall, severe COVID-19 cases were found to have higher level of ALAT (SMD = 0.53, 95%CI: 0.34;0,71; *P-value:* < 0.001; *n* = 2292) and ASAT (SMD = 0.96, 95%CI: 0.58;1.34; *P-value:* < 0.001; *n* = 2103), LDH (SMD = 1.36, 95%CI: 0.75;1.98; *P-value:* < 0.001; *n* = 1660), CK (SMD = 0.48, 95%CI: 0.10;0.87; *P-value:0.01*; n = 1660), total bilirubin (SMD = 0.32, 95%CI: 0.18;0.47; *P-value:* < 0.001; *n* = 1597), γ-GT (SMD = 1.03, 95%CI: 0.83;1.22;*P-value:*< 0.001; *n* = 616), myoglobin (SMD = 1.14, 95%CI: 0.81;1.47; *P-value:* < 0.001; *n* = 1012) and blood urea nitrogen (SMD = 0.32, 95%CI: 0.18;0.47; *P-value:*0.001; *n* = 1197) and Creatininemia (SMD = 0.18, 95%CI: 0.01;0.35; *P-value:* 0.04; *n* = 1785). Albuminemia was found to be lower in severe cases (SMD-1.67,95%CI -2.40; − 0.94; *P-value:< 0.001*; *n* = 1040) (Table 2).

Severe COVID-19 cases were more likely to have higher blood level of ASAT(RR = 2.27, 95%CI: 1.76;2.94; *P-value:*< 0.001; *n* = 1538), CK(RR = 2.61, 95%CI: 1.35;5.05; *P-value:* 0.01; *n* = 1086), Creatininemia (RR =3.66, 95%CI: 1.53; 8.81; *P-value:* 0.02; *n* = 1084), LDH (RR =2.03, 95%CI: 1.42;2.90; *P-value:* 0.003; *n* = 1138) (Table 4).

Concerning non-survivors, total bilirubin (SMD = 0.55,95%CI: 0.20;0.89; *P-value:* 0.002; *n* = 136) and LDH (SMD = 1.48,95%CI: 0.57;2.40; *P-value:* 0.002; *n* = 268) were significantly higher in non-survivors compare to survivors (Table 3).

## Discussion

This review shows that patients with a severe form of COVID-19 have higher level of CK-MB,troponin I, D-dimer, prothrombin time, procalcitonin, interleukin-6, C-reactive protein, ALAT, ASAT, LDH, CK, total bilirubin, γ-GT, myoglobin, blood urea nitrogen, creatininemia and lower level of lymphocyte, thrombocytes, eosinophils, haemoglobin and albuminemia.

### Inflammation and COVID-19

It has been shown that COVID-19 infection is associated with a severe systemic immune response drive by a “cytokine storm”. [42–44] Our data point to an association between procalcitonin (PCT), CRP levels and the severity of the disease. This is supported by previous findings which has reported elevated levels of several pro inflammatory cytokines including IL-2, IL-6, IL-7, MCP1, and TNF alpha, which contributes to increase classical inflammatory biomarkers such as PCT and CRP and are found to be higher in the severe forms of the disease. [42, 43, 45] Mechanistically, it has been show that, after the virus entry in the pneumocytes via the ACE2 receptor, it triggers a systemic inflammatory response, creating an immune dysfunction with a hyperactivity of T lymphocytes and the release of pro inflammatory cytokines mentioned above. [42, 43, 46] If the inflammation is initially protective, it appears secondarily that most cellular and tissue lesions are more the result of hyperinflammation than of the direct effect of the virus. [42, 43] These two phenomena may favor at the respiratory level, pulmonary lesions, by hypercapillary permeability and pulmonary edema, leading to acute respiratory distress. [42, 47] Hyperinflammation at the systemic level may lead to vascular, thrombotic and cytokines’ toxicity phenomena, resulting in multisystemic lesions. [43, 44, 48] Moore et al. showed that, these severe, multi-organ damage may worsen the prognosis despite supportive therapies. [49] Thus for clinicians, the measurement of classical and accessible inflammatory markers namely PCT and CRP, may help assessing the hyper inflammatory activity, and thus the severity of cases of severe infections. Furthermore, the use of therapies targeting inflammation to reduce the deleterious effects of the cytokine storm appear to be necessary. In this line, some studies have reported the beneficial effects of immunosuppressive and immunomodulatory therapies. [50–52] However, the benefit of the use of corticosteroids and non-steroidal anti-inflammatory drugs to reduce hyperinflammation during the 2019-nCoV infection is still controversial. [39–41]

### Cardiac biomarkers imbalanced and COVID-19

Our analysis revealed that severe COVID-19 cases have elevated levels of biomarkers of cardiomyocytes injury such as Troponin I, CK-MB, CK and myoglobin. Numerous previous researches have reported a cardiac involvement in patients with 2019-nCoV. [53–56] In a recently published study, Lippi and colleagues reported that the values of cardiac Troponin I were significantly increased in COVID-19 cases with severe disease than in those without. [55] Notwithstanding the incomplete knowledge on its pathophysiology, the mainly suggested mechanisms are: myocardial ischemia due to increased oxygen demand but in the context of hypoxemia and plaque disruption triggered by cytokine storm; these two being most frequently encountered among patients with coronary artery disease. [57–60] Also, myocardial inflammation, which can be the result of systemic immune response is another mechanism of heart injury among COVID-19 cases. [61, 62] Likewise, it has been hypothesised a direct viral toxicity through the interaction with ACE2 receptors highly expressed by some pericytes. [59] A thorough cardiac assessment should therefore be conducted in the follow-up of severe COVID-19 cases and manage properly to avoid adverse outcome.

### Lymphopenia and other full blood count imbalance in severe COVID-19 cases

Hematological changes encountered in the course of the novel coronavirus infection are common and concern mainly white blood cells and platelets. [63] In our study, some keys parameters modifications, notably lymphopenia and thrombocytopenia, are associated with severe form of the disease. [64]

Indeed, we found decreased overall mean levels of lymphocyte count among severe 2019-nCoV patients. Moreover, patients with severe 2019-nCoV infection were more likely to have lymphopenia. Low lymphocyte count has also been reported for other viral respiratory infection such as the one due to Respiratory Syncytial Virus (RSV). [65] This immune response marked by a profound lymphopenia seem to be is delay complication that come after an early massive release of cytokine during the course of the SRAS-Cov-2 lung injury. [66–68] The effects of COVID-19 on lymphocytes can be explained by direct and/or indirect mechanisms. The direct insult could be related to 2019-nCoV cytotoxicity, sustained by active viral replication within pool of infected lymphocytes. [67, 69] However, angiotensin-converting enzyme 2 (ACE2) has been identified as a functional cellular receptor for the COVID-19, a protein that is not expressed on circulating B or T lymphocytes. [66, 70] Studies suggested a potential role of alveolar macrophages which can promote viral entry via antigen presentation to T lymphocytes. [66, 67] Indirect lymphocytes damage could appear through huge cytokines release which can induce cells apoptosis. [66, 68, 69] In addition, high glucocorticoids levels induced by severe 2019-nCov can pertain to the down regulation of proinflammatory lymphokine ultimately lead to the lymphocyte activation and proliferation alteration . [67, 68]. [71] Clinicians should therefore keep in mind that severe cases of COVID-19 have depressed lymphocyte levels and are therefore more prone to infection.

Another key hematological disturbance found in our review was thrombocytopenia. Indeed, our analysis revealed significantly reduced mean platelets count in cases with severe COVID-19. Moreover, patients with severe form of the disease were more likely to have low platelets counts. Some potential mechanisms of SRAS-associated thrombocytopenia have been proposed. Coronavirus can infect bone marrow cells and thereby reduce platelet production. [72] Indeed some strains of coronavirus like HCoV-229 have their receptor on humans cells like those of kidney, lung and platelets, named Human amino peptidase N (CD13). [73] 2019-nCoV infection may also increase peripheral destruction of platelet [72, 74] by immunological mechanism by generating auto antibodies and immune complex. [75] Similar response have been reported for other viruses like HIV 1 in which Anti-platelet membrane GPIIIa49–66 IgG antibodies can cross-react with the HIV-1GP 160/120 antigen. [75] Other pathways can be the consumption of platelet following severe lung inflammation result in platelets activation and formation of microthrombi. [72] Thrombocytopenia, which makes cases with severe forms of the disease susceptible to primary coagulation impairment, is therefore an important element to consider and address in the daily management and evaluation of these patients.

### COVID-19 and blood coagulation

Four main coagulation biomarkers disturbances were found in severe COVID-19 cases namely higher serum D-dimers, longer prothrombin time (PT), and lower platelets counts. Moreover, we found that patients with severe 2019-nCoV infection are more likely to have increase serum D-dimers.

Coronavirus disease 2019 can affect haemostasis and blood coagulation in many ways, both regarding abnormal bleeding risk and thromboembolism. [72, 76–79] In addition to thrombocytopenia mechanistic hypotheses stated earlier, it must be suggest the potential implication of COVID-19 liver injury an aetiological element of haemostasis disorder though the impaired production of coagulation factors. [72, 76, 77]

Concerning D-dimers, increased levels might be the result of the sepsis-induced disseminated intravascular coagulation and reflect thromboembolic risk in severe COVID-19 cases. [80–82] There are mounting evidences supporting the key pathological role of thromboembolic processes in SARS-CoV-2 disease and severity. [83–86] Due to the low specificity of this biomarker, its results in Covid-19 patients should be interpreted in the context of other associated conditions that may also potentially increase D-dimer levels.

Decreased platelet counts and increased prothrombin time in patients with covid-19 are probably due to consumption coagulopathy. The latter occurs as a result of an abnormal increase in the activation of procoagulant pathways which induces a decrease in plasma concentrations of fibrinogen, platelets and other clotting factors. [87] A daily assessment of the coagulation profile of cases with severe forms of the disease appears vital and the correction of those abnormalities unavoidable to improve the prognosis. Cases of COVID-19 with blood-clotting abnormalities should be managed very closely and invasive treatments that may lead to bleeding should be avoided.

### Biochemical abnormalities in severe COVID-19 cases

Numerous liver biomarkers abnormalities have been described as being associated with COVID-19 encompassing increased levels of total bilirubin, transaminases, γ-GT, LDH, and low albumin levels. [88–91] Our results revealed that some of these biomarkers (ASAT/ALAT, γ-GT, LDH) have a significantly higher level in severe cases, and that severe cases are more likely to have high levels of ASAT and LDH.

It has been reported a virus-induced cytopathic effect notably through the binding with the ACE2 receptor for its target cells entry. [91, 92] Another potential triggering factor of detrimental liver consequences is immune-mediated inflammation in parallel with the “cytokine storm”. [76, 93, 94] Moreover, the immune responses ensured by B and T cells lymphocytes can also sustain this inflammation. [67, 68] It has also been put forward stress on sepsis related ischemic/hypoxic liver injury as putative mechanisms of abnormal hepatic function. [95, 96] Hepatic sinuses congestion related to thrombosis and/or high levels of positive end expiratory pressure can also be a contributing explanatory mechanisms of liver damage. [93, 95, 97] Likewise, COVID-19 patients may experience drug-induced liver injury (DILI), [93, 98] and reactivation of pre-existing hepatic conditions (going from viral hepatitis to liver cirrhosis). [90, 97, 99] Therefore, drugs which are known to be hepatotoxic or which are metabolized by the liver should be administered with caution or should be avoided in severe cases. Nevertheless, the results of liver biomarkers in COVID-19 cases must be interpreted in the context of the presence of other comorbidities that may induce an increase in these biomarkers.

Our findings point to an overall higher levels of urea nitrogen and creatinine among COVID-19 severe cases. Further studies are needed to better understand renal involvement in severe COVID-19 cases, especially since some pathophysiological hypotheses suggest the possibility of kidney damages related to COVID-19 infection. [100–103]

The current research provides evidence-based elements that can help clinicians in decision making when dealing with COVID-19 cases. Future studies should explore novel biomarkers, such as non-coding RNAs or proteomics, for disease progression and severity of COVID-19 and similar diseases in order to build powerful prediction tools.

The results of the current review should be interpreted in the context of some limitations. Firstly, it was not possible to assess the dynamic of biomarkers and to determine whether the samples were taken on admission or later during hospitalization. Therefore, the kinetic of biomarkers during the disease progression to severity cannot be established. Secondly, all the studies included in the meta-analysis were from China. Different biological patterns might be observed across populations from various geographical and ethnic background. Therefore, this limits the generalizability of our findings and call to more research on the topic in population from different countries and ethnicities. Thirdly, the measurement of biomarkers was probably different between studies, with various devices and diagnostic reference values. However, in most studies, there was low to moderate heterogeneity, suggesting that observations were consistent across studies. Nevertheless, this study is one of the first to analyse potential biomarkers associated with the severity of 2019-nCoV infection. We used state-of-the-art statistical methods in order to derive the most accurate estimates.

## Conclusion

Some inflammatory (procalcitonin, CRP), haematologic (lymphocyte, Thrombocytes), and biochemical (CK-MB, Troponin I, D-dimer, ASAT, ALAT, LDH, γ-GT) biomarkers are significantly associated with severe COVID-19. These biomarkers might help in prognostic risk stratification of patients with COVID-19. Our findings could also contribute in the establishment of an accurate and reproducible COVID-19 biological severity score.

## Supplementary information

**Additional file 1 Supplementary Table 1.** Search strategy in PubMed. **Supplementary Table 2.** Search strategy in EMBASE. **Supplementary Figure 1.** The review process.

## Data Availability

All related materials are available in the appendix.
