# Supplementary material for: A meta-analysis of potential biomarkers associated with severity of coronavirus disease 2019 (COVID-19)
Source: Biomark Res. 2020 Aug 31;8:37. doi: 10.1186/s40364-020-00217-0 (PMC7456766; doi:10.1186/s40364-020-00217-0)

A meta-analysis of potential prognostic biomarkers in coronavirus disease 2019 (2019-nCoV)

Celestin Danwang[1*]; Francky Teddy Endomba[2]; Jan René Nkeck[3]; Dominic Leandry Wouna Angong[4], Annie Robert[1], Jean Jacques Noubiap[5]

APPENDIX

Supplementary Figure 1. The review process 3

Supplementary table 1. Search strategy in PubMed and EMBASE 2
Supplementary table 2.Search strategy in EMBASE 2

I. Forest plot of studies reporting mean and standard deviation (or interquartile range) of biomarkers in severe and non-severe groups 4

Meta-analysis of routine blood biomarkers 4

Meta-analysis of Inflammation biomarkers 5

Meta-analysis of biochemical biomarkers 6

Meta-analysis of Blood clothing biomarkers 9

Meta-analysis of studies reporting survivor and non-survivors with mean of biomarkers . 10

II. Forest plot of studies reporting proportion of participants with each biomarker's abnormalities 12

Odds of blood routine abnormalities 12

Odds of inflammatory biomarkers abnormalities 13

Odds of blood clothing abnormalities 13

Odss of biochemical abnormalities 14

Supplementary table 1. Search strategy in PubMed and EMBASE

Search	Search terms
#1	COVID [tiab] OR "novel coronavirus"[tiab]
#2	Clinical features[tiab] OR clinical profil[tiab]
#3	blood disorders[MeSH Terms]
#4	#1 AND #2
#5	#1 AND #3
#6	#4 OR #5
Date: 18 April 2020 , restriction: 2019 and 2020	

Supplementary table 2.Search strategy in EMBASE

Search	Search terms
#1	covid OR 'novel coronavirus'
#2	clinical OR features OR profil
#3	blood AND disorders
#4	#1 AND #2
#5	#1 AND #3
#6	#4 OR #5
Date: 18 April 2020 , restriction: 2019 and 2020	

Supplementary Figure 1. The review process

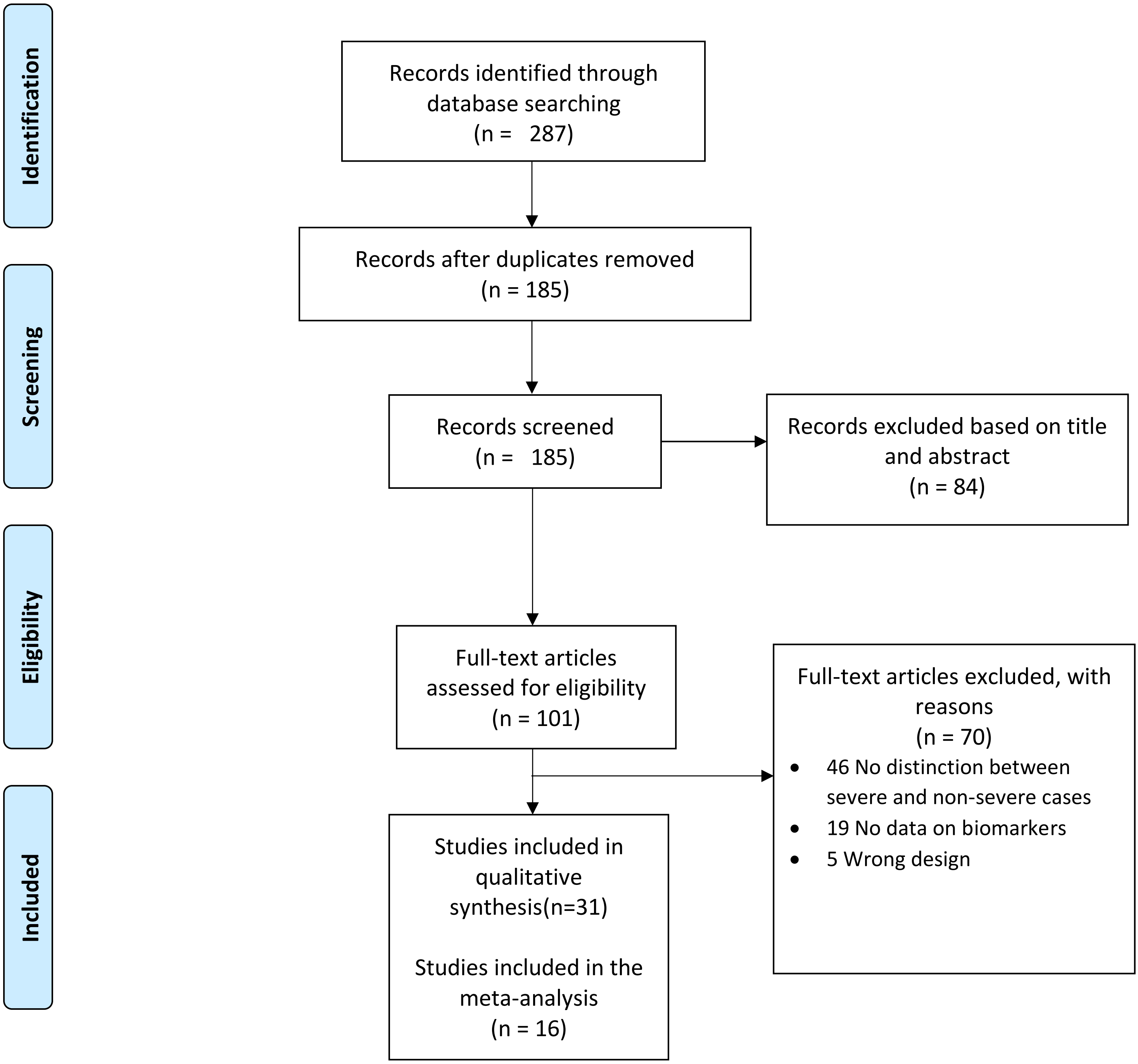

I. Forest plot of studies reporting mean and standard deviation (or interquartile range) of biomarkers in severe and non-severe groups

Meta-analysis of routine blood biomarkers

Lymphocytes count

Study	Severe Mean	Severe SD	Severe Total	Non-severe Mean	Non-severe SD	Non-severe Total	SMD	95%-CI	Weight
Wang,R	0.74	0.3111	25	1.11	0.4000	100	−0.96	[−1.41; −0.50]	6.0%
Feng,Y	0.82	0.4370	70	1.13	0.5481	352	−0.58	[−0.84; −0.32]	10.1%
Cai,Q	0.98	0.4000	58	1.34	0.6148	240	−0.62	[−0.91; −0.33]	9.3%
Wan,S	0.80	0.2963	40	1.20	0.5926	95	−0.76	[−1.14; −0.38]	7.3%
Li,K	0.70	0.3778	25	1.23	0.3630	58	−1.43	[−1.95; −0.91]	5.1%
Zheng,F	0.85	0.2741	30	1.12	0.4519	131	−0.63	[−1.04; −0.23]	6.9%
Guan,W	0.80	0.2963	153	1.00	0.4444	726	−0.47	[−0.65; −0.30]	12.2%
Zhang,J	0.70	0.3704	56	0.80	0.4444	82	−0.24	[−0.58; 0.10]	8.1%
Wang,D	0.80	0.2963	36	0.90	0.4444	102	−0.24	[−0.62; 0.14]	7.3%
Mao,L	0.90	1.8519	88	1.30	1.6296	126	−0.23	[−0.50; 0.04]	9.7%
Qin,C	0.80	0.3704	286	1.00	0.4444	166	−0.50	[−0.69; −0.31]	11.8%
Yun,L	0.78	0.4148	21	1.14	0.4741	271	−0.76	[−1.21; −0.32]	6.1%
**Random effects model**			**888**			**2449**	**−0.57**	**[−0.71; −0.42]**	**100.0%**

Heterogeneity: $I^2 = 61\%$, $\tau^2 = 0.0364$, $p < 0.01$

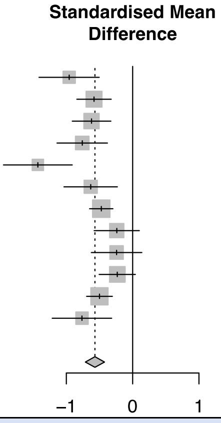

Lymphocytes %

Study	Severe Mean	Severe SD	Severe Total	Non-severe Mean	Non-severe SD	Non-severe Total	SMD	95%-CI	Weight
Li,K	13.20	6.2700	25	23.78	8.7200	58	−1.30	[−1.81; −0.79]	22.3%
Zhang,J	12.70	10.5926	56	20.00	11.7778	82	−0.64	[−0.99; −0.29]	32.6%
Qin,C	14.10	9.3333	286	21.40	12.7407	166	−0.68	[−0.88; −0.48]	45.1%
**Random effects model**			**367**			**306**	**−0.81**	**[−1.12; −0.49]**	**100.0%**

Heterogeneity: $I^2 = 62\%$, $\tau^2 = 0.0466$, $p = 0.07$

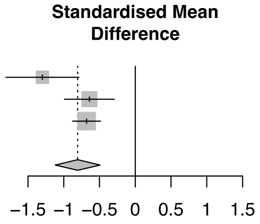

Thrombocytes

Study	Severe Mean	Severe SD	Severe Total	Non-severe Mean	Non-severe SD	Non-severe Total	SMD	95%-CI	Weight
Wang,R	163.40	60.2600	25	169.50	51.8519	100	−0.11	[−0.55; 0.33]	11.4%
Feng,Y	181.00	82.2222	70	185.00	68.1481	352	−0.06	[−0.31; 0.20]	16.2%
Wan,S	147.00	70.3704	40	170.00	72.5926	95	−0.32	[−0.69; 0.05]	13.1%
Zheng,F	160.00	60.0000	30	171.00	62.2222	131	−0.18	[−0.57; 0.22]	12.4%
Guan,W	137.50	59.6296	156	172.00	54.0741	713	−0.63	[−0.80; −0.45]	18.4%
Wang,D	142.00	61.4815	36	165.00	46.6667	102	−0.45	[−0.83; −0.07]	12.7%
Mao,L	204.50	413.3333	88	219.00	400.7407	126	−0.04	[−0.31; 0.24]	15.8%
**Random effects model**			**445**			**1619**	**−0.26**	**[−0.48; −0.04]**	**100.0%**

Heterogeneity: $I^2 = 72\%$, $\tau^2 = 0.0599$, $p < 0.01$

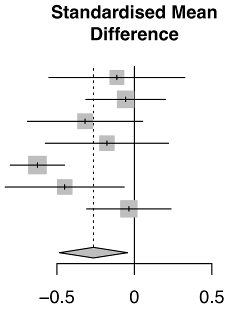

Eosinophils

Study	Severe Mean	Severe SD	Severe Total	Non-severe Mean	Non-severe SD	Non-severe Total	SMD	95%-CI	Weight
Cai,Q	0.01	0.0222	58	0.02	0.0370	240	−0.29	[−0.58; 0.00]	58.5%
Zhang,J	0.01	0.0444	56	0.02	0.0311	82	−0.27	[−0.61; 0.07]	41.5%
**Random effects model**			**114**			**322**	**−0.28**	**[−0.50; −0.06]**	**100.0%**

Heterogeneity: $I^2 = 0\%$, $\tau^2 = 0$, $p = 0.93$

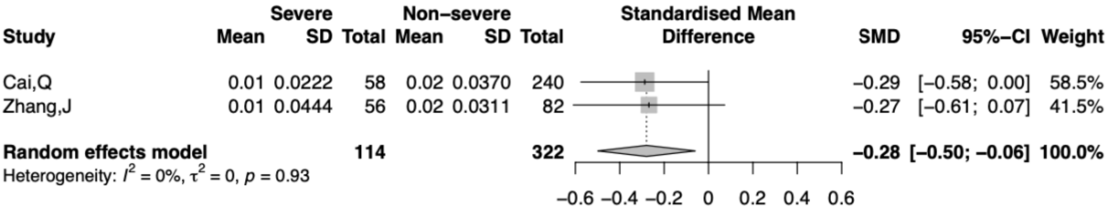

Neutrophils

Study	Severe Mean	Severe SD	Severe Total	Non-severe Mean	Non-severe SD	Non-severe Total	SMD	95%-CI	Weight
Wang,R	3.86	2.1800	25	3.49	1.6200	100	0.21	[−0.23; 0.65]	9.8%
Feng,Y	5.99	4.5037	70	3.39	1.5852	352	1.11	[0.85; 1.38]	12.4%
Cai,Q	7.35	3.1111	58	6.65	2.5185	240	0.26	[−0.02; 0.55]	12.1%
Wan,S	4.10	1.9259	40	3.60	0.6667	95	0.42	[0.05; 0.79]	10.8%
Li,K	4.36	2.6741	25	3.50	1.3481	58	0.46	[−0.01; 0.94]	9.3%
Wang,D	4.60	3.9259	36	2.70	1.4815	102	0.80	[0.41; 1.19]	10.5%
Mao,L	3.80	13.8519	88	2.60	8.2222	126	0.11	[−0.16; 0.38]	12.3%
Qin,C	4.30	3.0370	286	3.20	1.7037	166	0.42	[0.22; 0.61]	13.3%
Yun,L	4.06	1.2667	21	3.18	0.9111	271	0.93	[0.48; 1.38]	9.6%
**Random effects model**			**649**			**1510**	**0.52**	**[0.28; 0.76]**	**100.0%**

Heterogeneity: $I^2 = 80\%$, $\tau^2 = 0.1013$, $p < 0.01$

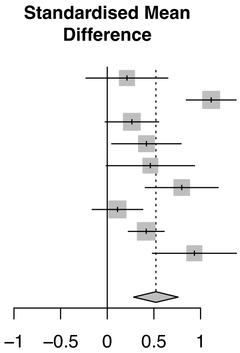

Haemoglobin

Study	Severe Mean	Severe SD	Severe Total	Non-severe Mean	Non-severe SD	Non-severe Total	SMD	95%-CI	Weight
Wang,R	136.68	16.9200	25	140.86	15.2700	100	−0.27	[−0.71; 0.17]	15.2%
Feng,Y	131.00	18.5185	70	133.00	17.0370	352	−0.12	[−0.37; 0.14]	44.7%
Wan,S	130.00	17.0370	40	134.00	17.0370	95	−0.23	[−0.60; 0.14]	21.5%
Zheng,F	126.00	17.0370	30	131.00	14.8148	131	−0.33	[−0.72; 0.07]	18.6%
**Random effects model**			**165**			**678**	**−0.20**	**[−0.37; −0.03]**	**100.0%**

Heterogeneity: $I^2 = 0\%$, $\tau^2 = 0$, $p = 0.82$

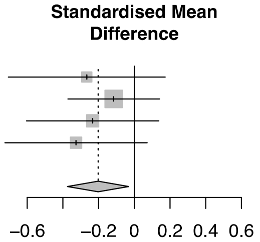

Monocytes

Study	Severe Mean	Severe SD	Severe Total	Non-severe Mean	Non-severe SD	Non-severe Total	SMD	95%-CI	Weight
Wang,R	0.30	0.2111	25	0.36	0.1852	100	−0.31	[−0.75; 0.13]	14.3%
Li,K	0.34	0.2667	25	0.42	0.1778	58	−0.38	[−0.85; 0.09]	12.5%
Wang,D	0.40	0.1481	36	0.40	0.1481	102	0.00	[−0.38; 0.38]	18.6%
Qin,C	0.40	0.1481	286	0.40	0.1481	166	0.00	[−0.19; 0.19]	54.6%
**Random effects model**			**372**			**426**	**−0.09**	**[−0.27; 0.08]**	**100.0%**

Heterogeneity: $I^2 = 14\%$, $\tau^2 = 0.0050$, $p = 0.32$

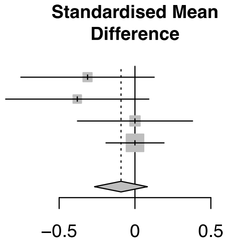

White Blood Cells

Study	Severe Mean	Severe SD	Severe Total	Non-severe Mean	Non-severe SD	Non-severe Total	SMD	95%-CI	Weight
Wang,R	5.11	2.3200	25	4.92	1.6793	100	0.10	[−0.33; 0.54]	8.2%
Feng,Y	5.15	1.7556	351	5.15	1.7556	351	0.00	[−0.15; 0.15]	10.3%
Cai,Q	4.50	1.8519	58	4.53	1.4926	240	−0.02	[−0.30; 0.27]	9.5%
Wan,S	5.20	1.4815	40	5.50	2.9630	95	−0.11	[−0.48; 0.26]	8.8%
Li,K	5.50	2.6667	25	5.27	1.5556	58	0.12	[−0.35; 0.59]	8.0%
Zheng,F	4.76	1.8963	30	4.17	1.4148	131	0.39	[−0.01; 0.79]	8.6%
Guan,W	3.70	2.3711	173	4.90	1.6304	926	−0.68	[−0.84; −0.51]	10.2%
Wang,D	6.60	4.5926	36	4.30	1.5556	102	0.85	[0.46; 1.24]	8.6%
Mao,L	5.40	15.0370	88	4.50	9.0370	126	0.08	[−0.20; 0.35]	9.6%
Qin,C	5.60	3.0370	286	4.90	1.7778	166	0.26	[0.07; 0.46]	10.1%
Yun,L	5.85	4.3778	21	4.75	1.4889	271	0.60	[0.15; 1.04]	8.1%
**Random effects model**			**1133**			**2566**	**0.13**	**[−0.14; 0.39]**	**100.0%**

Heterogeneity: $I^2 = 90\%$, $\tau^2 = 0.1668$, $p < 0.01$

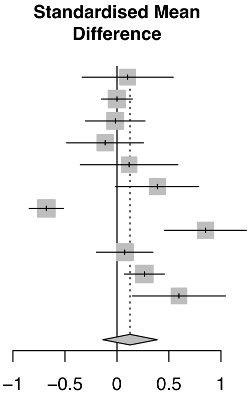

Meta-analysis of Inflammation biomarkers

Procalcitonin

Study	Severe Mean	Severe SD	Severe Total	Non−severe Mean	Non−severe SD	Non−severe Total	SMD	95%−CI	Weight
Wang,R	0.05	0.0867	25	0.03	0.0311	100	0.50	[0.05; 0.94]	15.8%
Feng,Y	0.07	0.1333	70	0.04	0.0296	352	0.50	[0.24; 0.75]	18.3%
Wan,S	0.11	0.0593	40	0.04	0.0222	95	1.87	[1.44; 2.31]	15.9%
Li,K	0.09	0.1193	25	0.04	0.0296	58	0.68	[0.20; 1.17]	15.2%
Qin,C	0.10	0.1481	286	0.05	0.0444	166	0.41	[0.22; 0.61]	19.0%
Yun,L	0.10	0.5852	21	0.03	0.0222	271	0.45	[0.00; 0.90]	15.8%
**Random effects model**			**467**			**1042**	**0.72**	**[0.34; 1.11]**	**100.0%**

Heterogeneity: $I^2 = 87\%$, $\tau^2 = 0.1934$, $p < 0.01$

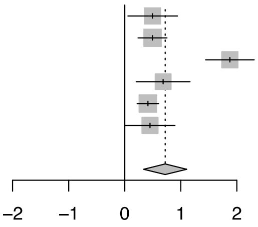

C-reactive protein

Study	Severe Mean	Severe SD	Severe Total	Non−severe Mean	Non−severe SD	Non−severe Total	SMD	95%−CI	Weight
Wang,R	56.26	47.8800	25	10.80	16.6111	100	1.75	[1.26; 2.24]	10.7%
Feng,Y	83.40	72.5926	63	12.00	24.5926	307	1.91	[1.61; 2.22]	11.4%
Cai,Q	32.23	29.4667	58	8.18	13.4963	240	1.35	[1.05; 1.66]	11.4%
Wan,S	91.00	61.9259	40	7.70	21.6296	95	2.17	[1.72; 2.62]	10.9%
Li,K	89.20	64.2667	25	9.59	20.6074	58	2.02	[1.46; 2.59]	10.4%
Zheng,F	52.20	34.2963	30	15.40	14.1481	131	1.88	[1.44; 2.33]	10.9%
Zhang,J	47.60	49.2593	55	28.70	31.5556	81	0.47	[0.13; 0.82]	11.3%
Mao,L	37.10	156.9630	88	9.40	93.0370	126	0.22	[−0.05; 0.50]	11.5%
Qin,C	57.90	60.9630	286	32.20	38.1481	166	0.48	[0.28; 0.67]	11.7%
**Random effects model**			**670**			**1304**	**1.34**	**[0.83; 1.86]**	**100.0%**

Heterogeneity: $I^2 = 95\%$, $\tau^2 = 0.5801$, $p < 0.01$

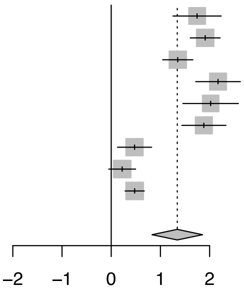

Interleukin-6

Study	Severe Mean	Severe SD	Severe Total	Non−severe Mean	Non−severe SD	Non−severe Total	SMD	95%−CI	Weight
Wang,R	39.80	27.2963	25	16.80	17.8889	100	1.14	[0.68; 1.60]	31.0%
Cai,Q	26.95	25.2667	58	8.51	9.3704	240	1.32	[1.02; 1.63]	33.7%
Qin,C	25.20	33.2593	286	13.30	27.5556	166	0.38	[0.19; 0.57]	35.2%
**Random effects model**			**369**			**506**	**0.93**	**[0.25; 1.61]**	**100.0%**

Heterogeneity: $I^2 = 93\%$, $\tau^2 = 0.3312$, $p < 0.01$

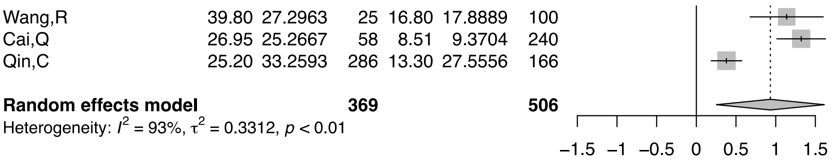

Erythrocytes sedimentation rate

Study	Severe Mean	Severe SD	Severe Total	Non−severe Mean	Non−severe SD	Non−severe Total	SMD	95%−CI	Weight
Feng,Y	58.00	24.4444	70	48.00	41.4815	352	0.25	[0.00; 0.51]	25.9%
Cai,Q	45.00	24.4444	58	24.00	21.4815	240	0.95	[0.65; 1.25]	25.2%
Qin,C	34.00	30.3704	286	28.00	26.6667	166	0.21	[0.01; 0.40]	27.0%
Yun,L	52.00	33.3333	21	70.00	45.5556	271	−0.40	[−0.85; 0.04]	21.9%
**Random effects model**			**435**			**1029**	**0.27**	**[−0.16; 0.70]**	**100.0%**

Heterogeneity: $I^2 = 90\%$, $\tau^2 = 0.1700$, $p < 0.01$

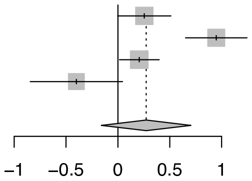

Meta-analysis of biochemical biomarkers

ALAT

Study	Severe Mean	Severe SD	Severe Total	Non-severe Mean	Non-severe SD	Non-severe Total	SMD	95%-CI	Weight
Cai,Qi	67.00	39.2593	85	41.00	31.1111	233	0.77	[0.52; 1.03]	11.8%
Wang,R	25.00	12.9630	25	24.00	18.3333	100	0.06	[−0.38; 0.50]	8.2%
Feng,Y	35.00	20.7407	70	23.00	17.0370	352	0.68	[0.42; 0.94]	11.7%
Cai,Q	26.85	15.4074	58	20.00	11.2963	240	0.56	[0.27; 0.85]	11.1%
Wan,S	26.60	13.9259	40	21.70	16.3704	95	0.31	[−0.06; 0.68]	9.4%
Zheng,F	23.90	13.1111	30	19.30	2.3704	131	0.76	[0.36; 1.17]	8.8%
Zhou,F	40.00	20.0000	54	27.00	18.5185	135	0.68	[0.36; 1.01]	10.4%
Wang,D	35.00	28.1481	36	23.00	15.5556	102	0.61	[0.22; 1.00]	9.2%
Mao,L	32.50	1428.1481	88	23.00	188.8889	126	0.01	[−0.26; 0.28]	11.4%
Yun,L	32.00	24.4444	21	21.00	12.5926	271	0.80	[0.35; 1.25]	8.1%
**Random effects model**			**507**			**1785**	**0.53**	**[0.34; 0.71]**	**100.0%**

Heterogeneity: $I^2 = 68\%$, $\tau^2 = 0.0597$, $p < 0.01$

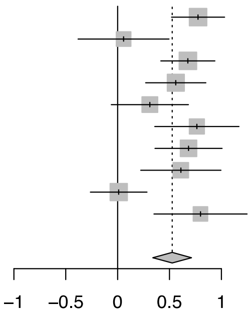

ASAT

Study	Severe Mean	Severe SD	Severe Total	Non-severe Mean	Non-severe SD	Non-severe Total	SMD	95%-CI	Weight
Cai,Qi	58.00	38.5185	85	34.00	13.3333	233	1.05	[0.78; 1.31]	11.7%
Wang,R	29.00	9.6296	25	26.00	10.3704	100	0.29	[−0.15; 0.73]	10.6%
Feng,Y	39.00	17.7778	70	25.00	11.1111	352	1.12	[0.85; 1.39]	11.7%
Cai,Q	36.00	15.0370	58	26.00	9.6296	240	0.92	[0.62; 1.21]	11.5%
Wan,S	33.60	13.7037	40	22.40	10.0741	95	0.99	[0.60; 1.38]	11.0%
Zheng,F	31.60	17.3778	30	23.40	7.2593	131	0.82	[0.42; 1.23]	10.8%
Wang,D	52.00	29.6296	36	29.00	12.5926	102	1.23	[0.83; 1.64]	10.8%
Mao,L	34.00	6061.4815	88	23.00	174.0741	126	0.00	[−0.27; 0.28]	11.6%
Yun,L	47.00	20.7407	21	23.00	8.8889	271	2.36	[1.87; 2.84]	10.3%
**Random effects model**			**453**			**1650**	**0.96**	**[0.58; 1.34]**	**100.0%**

Heterogeneity: $I^2 = 91\%$, $\tau^2 = 0.3033$, $p < 0.01$

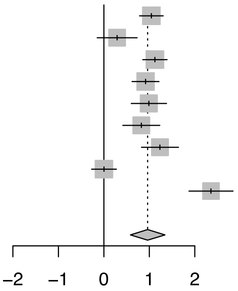

Albumin

Study	Severe Mean	Severe SD	Severe Total	Non-severe Mean	Non-severe SD	Non-severe Total	SMD	95%-CI	Weight
Feng,Y	32.25	4.7926	70	39.14	5.5926	352	−1.26	[−1.53; −0.99]	26.1%
Wan,S	36.00	4.0741	40	49.90	4.5926	95	−3.11	[−3.64; −2.58]	23.7%
Zhou,F	29.10	3.5556	54	33.60	4.2963	137	−1.09	[−1.43; −0.76]	25.6%
Yun,L	35.80	4.6000	21	40.90	3.8000	271	−1.32	[−1.77; −0.86]	24.5%
**Random effects model**			**185**			**855**	**−1.67**	**[−2.40; −0.94]**	**100.0%**

Heterogeneity: $I^2 = 93\%$, $\tau^2 = 0.5095$, $p < 0.01$

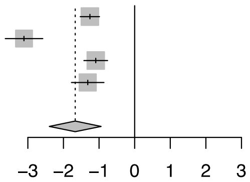

CK

Study	Severe Mean	Severe SD	Severe Total	Non-severe Mean	Non-severe SD	Non-severe Total	SMD	95%-CI	Weight
Feng,Y	93.00	143.7037	70	80.00	61.4815	352	0.16	[−0.10; 0.42]	15.2%
Cai,Q	87.00	88.0741	58	64.50	34.8148	240	0.45	[0.16; 0.74]	14.9%
Wan,S	82.00	66.5926	40	57.00	37.0370	95	0.52	[0.15; 0.90]	14.1%
Zheng,F	100.30	249.8519	30	68.70	50.4444	131	0.27	[−0.13; 0.67]	13.8%
Wang,D	102.00	140.7407	36	87.00	49.6296	102	0.18	[−0.20; 0.56]	14.0%
Mao,L	83.00	9042.3704	88	59.00	919.2593	126	0.00	[−0.27; 0.28]	15.0%
Yun,L	227.00	236.2963	21	77.50	45.7778	271	1.96	[1.49; 2.43]	13.0%
**Random effects model**			**343**			**1317**	**0.48**	**[0.10; 0.87]**	**100.0%**

Heterogeneity: $I^2 = 89\%$, $\tau^2 = 0.2342$, $p < 0.01$

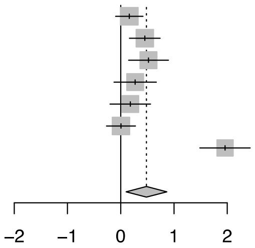

CK-MB

Study	Severe Mean	Severe SD	Severe Total	Non-severe Mean	Non-severe SD	Non-severe Total	SMD	95%-CI	Weight
Feng,Y	15.50	8.3333	70	12.75	4.3556	352	0.53	[0.27; 0.78]	34.5%
Cai,Q	1.13	0.8593	58	0.76	0.3556	240	0.75	[0.45; 1.04]	29.6%
Wang,D	18.00	17.0370	36	13.00	2.9630	102	0.55	[0.17; 0.94]	20.1%
Yun,L	17.10	10.4444	21	12.50	3.6296	271	1.03	[0.58; 1.48]	15.7%
**Random effects model**			**185**			**965**	**0.68**	**[0.48; 0.87]**	**100.0%**

Heterogeneity: $I^2 = 30\%$, $\tau^2 = 0.0122$, $p = 0.23$

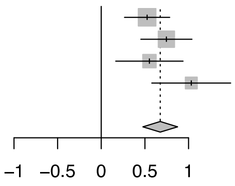

Troponin

Study	Severe Mean	Severe SD	Severe Total	Non-severe Mean	Non-severe SD	Non-severe Total	SMD	95%-CI	Weight
Wang,D	11.00	15.4074	36	5.10	5.7037	102	0.64	[0.25; 1.02]	57.3%
Yun,L	0.04	0.0459	21	0.02	0.0207	271	0.81	[0.36; 1.26]	42.7%
**Random effects model**			**57**			**373**	**0.71**	**[0.42; 1.00]**	**100.0%**

Heterogeneity: $I^2 = 0\%$, $\tau^2 = 0$, $p = 0.56$

Standardised Mean Difference

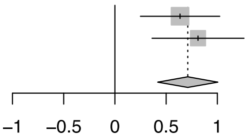

Creatinemia

Study	Severe Mean	Severe SD	Severe Total	Non-severe Mean	Non-severe SD	Non-severe Total	SMD	95%-CI	Weight
Wang,R	67.32	16.8100	25	64.50	15.9970	100	0.17	[−0.27; 0.61]	9.6%
Feng,Y	67.95	19.2963	70	65.46	17.5556	352	0.14	[−0.12; 0.40]	16.5%
Cai,Q	72.00	31.8519	58	61.00	17.7778	240	0.52	[0.23; 0.81]	14.9%
Wan,S	63.50	16.8889	40	66.00	17.7778	95	−0.14	[−0.51; 0.23]	11.7%
Zheng,F	47.50	18.5185	30	48.30	14.1481	131	−0.05	[−0.45; 0.34]	10.8%
Wang,D	80.00	29.6296	36	71.00	19.2593	102	0.40	[0.02; 0.78]	11.3%
Mao,L	71.60	6962.2963	88	65.60	140.5185	126	0.00	[−0.27; 0.27]	15.7%
Yun,L	72.30	43.8519	21	63.00	18.2222	271	0.44	[0.00; 0.89]	9.4%
**Random effects model**			**368**			**1417**	**0.18**	**[0.01; 0.35]**	**100.0%**

Heterogeneity: $I^2 = 49\%$, $\tau^2 = 0.0290$, $p = 0.06$

Standardised Mean Difference

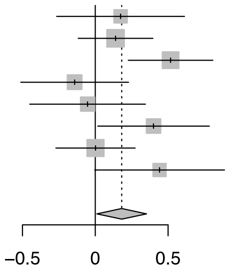

Blood urea nitrogen

Study	Severe Mean	Severe SD	Severe Total	Non-severe Mean	Non-severe SD	Non-severe Total	SMD	95%-CI	Weight
Wang,R	4.61	2.2800	25	3.90	1.2000	100	0.48	[0.03; 0.92]	17.6%
Feng,Y	5.65	2.5407	70	4.60	1.4741	352	0.62	[0.36; 0.88]	21.5%
Cai,Q	5.20	1.8889	58	3.84	1.2074	240	0.99	[0.69; 1.29]	20.8%
Wang,D	5.90	3.9259	36	4.00	1.4815	102	0.80	[0.41; 1.19]	18.7%
Mao,L	4.60	34.5185	88	3.80	8.9630	126	0.03	[−0.24; 0.31]	21.3%
**Random effects model**			**277**			**920**	**0.58**	**[0.23; 0.93]**	**100.0%**

Heterogeneity: $I^2 = 83\%$, $\tau^2 = 0.1321$, $p < 0.01$

Standardised Mean Difference

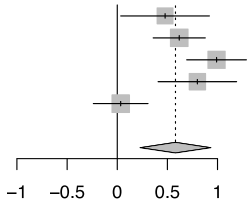

Total bilirubin

Study	Severe Mean	Severe SD	Severe Total	Non-severe Mean	Non-severe SD	Non-severe Total	SMD	95%-CI	Weight
Cai,Qi	22.00	7.4074	85	19.00	9.6296	233	0.33	[0.08; 0.58]	20.5%
Wang,R	10.40	8.0741	25	9.25	5.5741	100	0.19	[−0.25; 0.62]	9.1%
Feng,Y	12.20	6.0000	70	9.50	4.4444	352	0.57	[0.31; 0.83]	19.6%
Cai,Q	11.25	7.3333	58	10.90	5.5556	240	0.06	[−0.23; 0.35]	17.2%
Wan,S	9.80	5.7778	40	8.60	6.2222	95	0.20	[−0.17; 0.57]	11.9%
Zheng,F	12.70	5.7037	30	10.70	5.2741	131	0.37	[−0.03; 0.77]	10.6%
Wang,D	11.50	6.6667	36	9.30	3.4074	102	0.49	[0.10; 0.87]	11.2%
**Random effects model**			**344**			**1253**	**0.32**	**[0.18; 0.47]**	**100.0%**

Heterogeneity: $I^2 = 28\%$, $\tau^2 = 0.0106$, $p = 0.22$

Standardised Mean Difference

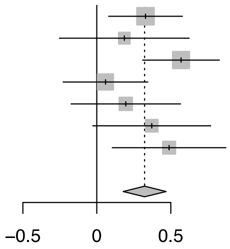

LDH

Study	Severe Mean	Severe SD	Severe Total	Non-severe Mean	Non-severe SD	Non-severe Total	SMD	95%-CI	Weight
Feng,Y	378.00	183.7037	70	236.00	90.3704	352	1.27	[1.00; 1.55]	14.7%
Cai,Q	387.00	273.3333	58	216.00	132.5926	240	1.01	[0.71; 1.31]	14.6%
Wan,S	309.00	114.4444	40	212.00	58.8889	95	1.22	[0.82; 1.61]	14.2%
Zheng,F	226.20	90.0741	30	162.00	55.4074	131	1.01	[0.60; 1.42]	14.2%
Wang,D	435.00	217.7778	36	212.00	88.8889	102	1.65	[1.22; 2.08]	14.1%
Mao,L	302.00	650.2222	88	215.00	670.7407	126	0.13	[−0.14; 0.40]	14.7%
Yun,L	454.00	138.5185	21	224.00	58.8889	271	3.40	[2.88; 3.92]	13.6%
**Random effects model**			**343**			**1317**	**1.36**	**[0.75; 1.98]**	**100.0%**

Heterogeneity: $I^2 = 95\%$, $\tau^2 = 0.6609$, $p < 0.01$

Standardised Mean Difference

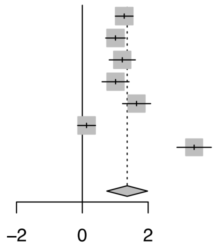

Myoglobin

Study	Severe Mean	Severe SD	Severe Total	Non-severe Mean	Non-severe SD	Non-severe Total	SMD	95%-CI	Weight
Feng,Y	52.05	57.6519	70	11.70	27.0741	352	1.18	[0.91; 1.45]	38.2%
Cai,Q	67.90	49.5926	58	33.52	12.2741	240	1.41	[1.10; 1.71]	35.4%
Yun,L	32.10	133.0370	21	5.90	7.3333	271	0.73	[0.29; 1.18]	26.4%
**Random effects model**			**149**			**863**	**1.14**	**[0.81; 1.47]**	**100.0%**

Heterogeneity: $I^2 = 66\%$, $\tau^2 = 0.0554$, $p = 0.05$

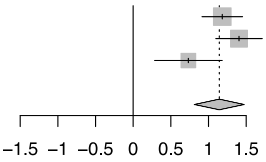

Potassium

Study	Severe Mean	Severe SD	Severe Total	Non-severe Mean	Non-severe SD	Non-severe Total	SMD	95%-CI	Weight
Feng,Y	4.00	0.6667	70	3.90	0.3704	352	0.23	[−0.03; 0.49]	34.2%
Wan,S	3.80	0.5926	40	4.00	0.5926	95	−0.34	[−0.71; 0.04]	27.9%
Guan,W	3.80	0.4444	138	3.90	0.4444	614	−0.22	[−0.41; −0.04]	37.9%
**Random effects model**			**248**			**1061**	**−0.10**	**[−0.43; 0.23]**	**100.0%**

Heterogeneity: $I^2 = 79\%$, $\tau^2 = 0.0671$, $p < 0.01$

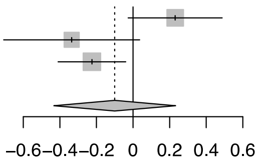

Sodium

Study	Severe Mean	Severe SD	Severe Total	Non-severe Mean	Non-severe SD	Non-severe Total	SMD	95%-CI	Weight
Feng,Y	140.00	3.7037	70	139.00	2.9630	352	0.32	[0.06; 0.58]	33.9%
Wan,S	136.50	3.7037	40	139.00	2.8148	95	−0.80	[−1.18; −0.42]	31.0%
Guan,W	138.00	2.9630	121	138.40	2.8148	536	−0.14	[−0.34; 0.06]	35.1%
**Random effects model**			**231**			**983**	**−0.19**	**[−0.72; 0.34]**	**100.0%**

Heterogeneity: $I^2 = 91\%$, $\tau^2 = 0.1992$, $p < 0.01$

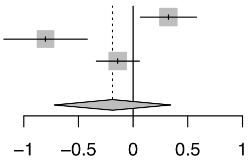

γ-GT

Study	Severe Mean	Severe SD	Severe Total	Non-severe Mean	Non-severe SD	Non-severe Total	SMD	95%-CI	Weight
Cai,Qi	92.00	80.0000	85	40.00	26.6667	233	1.10	[0.84; 1.36]	56.0%
Cai,Q	35.25	22.5185	58	21.00	12.8519	240	0.94	[0.64; 1.23]	44.0%
**Random effects model**			**143**			**473**	**1.03**	**[0.83; 1.22]**	**100.0%**

Heterogeneity: $I^2 = 0\%$, $\tau^2 = 0$, $p = 0.41$

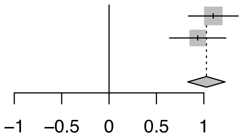

Meta-analysis of Blood clothing biomarkers

Prothrombin time

Study	Severe Mean	Severe SD	Severe Total	Non-severe Mean	Non-severe SD	Non-severe Total	SMD	95%-CI	Weight
Wan,S	11.30	0.8148	40	10.80	0.6667	95	0.70	[0.32; 1.08]	36.1%
Wang,D	13.20	1.6296	36	12.90	0.8148	102	0.28	[−0.11; 0.66]	35.7%
Han,H	12.65	1.1300	35	12.20	0.8800	49	0.45	[0.01; 0.89]	28.1%
**Random effects model**			**111**			**246**	**0.48**	**[0.23; 0.73]**	**100.0%**

Heterogeneity: $I^2 = 16\%$, $\tau^2 = 0.0080$, $p = 0.30$

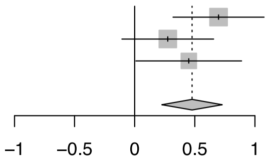

D-dimer

Study	Severe Mean	Severe SD	Severe Total	Non−severe Mean	Non−severe SD	Non−severe Total	SMD	95%−CI	Weight
Feng,Y	1.11	2.5852	70	0.51	0.5630	352	0.51	[0.25; 0.77]	16.9%
Cai,Q	0.56	0.4222	58	0.36	0.2000	240	0.77	[0.48; 1.07]	15.9%
Wan,S	0.60	0.5185	40	0.30	0.2222	95	0.88	[0.50; 1.27]	13.5%
Wang,D	414.00	839.2593	36	166.00	136.2963	102	0.56	[0.17; 0.94]	13.4%
Han,H	19.11	35.4800	35	2.14	2.8800	49	0.73	[0.28; 1.18]	11.9%
Mao,L	0.90	14.7407	88	0.40	6.2963	126	0.05	[−0.23; 0.32]	16.5%
Yun,L	1.00	6.0815	21	0.41	0.3037	271	0.36	[−0.08; 0.81]	12.0%
**Random effects model**			**348**			**1235**	**0.54**	**[0.31; 0.77]**	**100.0%**

Heterogeneity: $I^2 = 69\%$, $\tau^2 = 0.0654$, $p < 0.01$

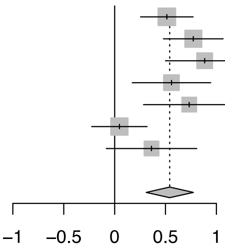

Activated partial thromboplastin

Study	Severe Mean	Severe SD	Severe Total	Non−severe Mean	Non−severe SD	Non−severe Total	SMD	95%−CI	Weight
Wan,S	29.70	12.4296	40	26.60	3.1852	95	0.43	[0.05; 0.80]	26.3%
Huang,C	29.53	3.4800	53	27.70	6.8889	28	0.37	[−0.09; 0.83]	23.5%
Wang,D	30.40	4.0741	36	31.70	2.8889	102	−0.40	[−0.78; −0.02]	26.0%
Han,H	29.53	3.4800	35	28.56	2.6600	49	0.32	[−0.12; 0.75]	24.3%
**Random effects model**			**164**			**274**	**0.17**	**[−0.23; 0.57]**	**100.0%**

Heterogeneity: $I^2 = 74\%$, $\tau^2 = 0.1234$, $p < 0.01$

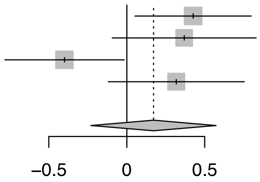

Fibrinogen

Study	Severe Mean	Severe SD	Severe Total	Non−severe Mean	Non−severe SD	Non−severe Total	SMD	95%−CI	Weight
Han,H	4.76	1.7301	35	5.10	1.1600	49	−0.24	[−0.67; 0.20]	50.3%
Yun,L	4.72	1.0074	21	4.26	1.0815	271	0.43	[−0.02; 0.87]	49.7%
**Random effects model**			**56**			**320**	**0.09**	**[−0.56; 0.74]**	**100.0%**

Heterogeneity: $I^2 = 77\%$, $\tau^2 = 0.1691$, $p = 0.04$

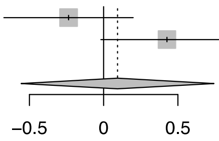

Meta-analysis of studies reporting survivor and non-survivors with mean of biomarkers

Lymphocytes

Study	Non−survivor Mean	Non−survivor SD	Non−survivor Total	Survivor Mean	Survivor SD	Survivor Total	SMD	95%−CI	Weight
Zhou,F	0.60	0.2222	54	1.10	0.5185	137	−1.09	[−1.43; −0.76]	37.5%
Wu,C	0.59	0.1926	44	0.80	0.4519	40	−0.61	[−1.05; −0.17]	33.5%
Yang,X	0.62	0.3700	32	0.70	0.4000	20	−0.21	[−0.77; 0.35]	28.9%
**Random effects model**			**130**			**197**	**−0.67**	**[−1.18; −0.17]**	**100.0%**

Heterogeneity: $I^2 = 75\%$, $\tau^2 = 0.1476$, $p = 0.02$

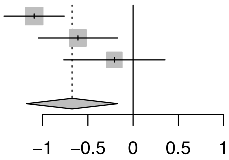

CD3+ T

Study	Severe Mean	Severe SD	Severe Total	Non−severe Mean	Non−severe SD	Non−severe Total	SMD	95%−CI	Weight
Qin,C	461.60	264.7000	286	663.80	291.3000	166	−0.73	[−0.93; −0.54]	83.9%
Yun,L	421.00	311.8519	21	776.00	369.0370	271	−0.97	[−1.42; −0.52]	16.1%
**Random effects model**			**307**			**437**	**−0.77**	**[−0.95; −0.59]**	**100.0%**

Heterogeneity: $I^2 = 0\%$, $\tau^2 = 0$, $p = 0.35$

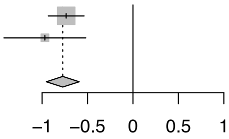

White blood cells

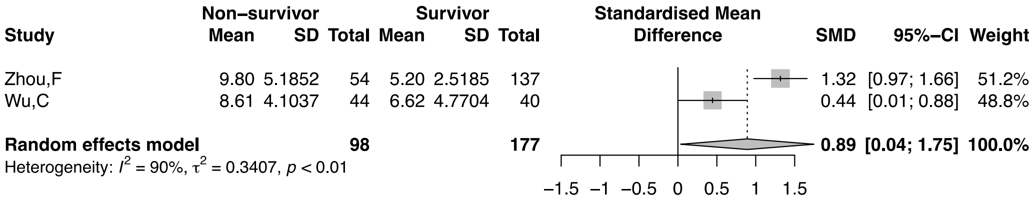

Interleukin-6

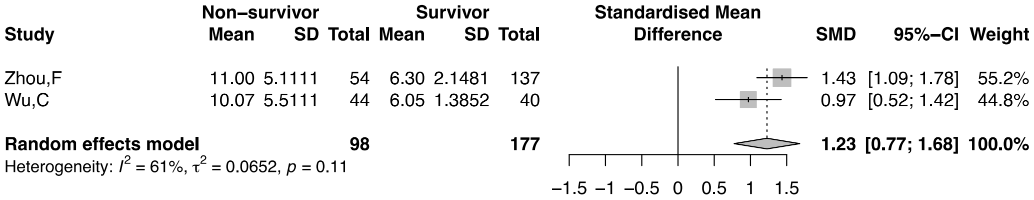

TP

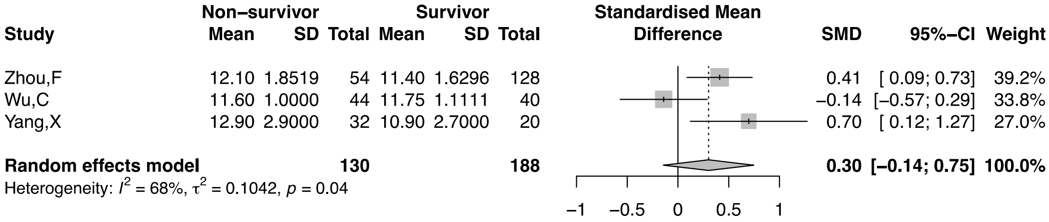

Total bilirubin

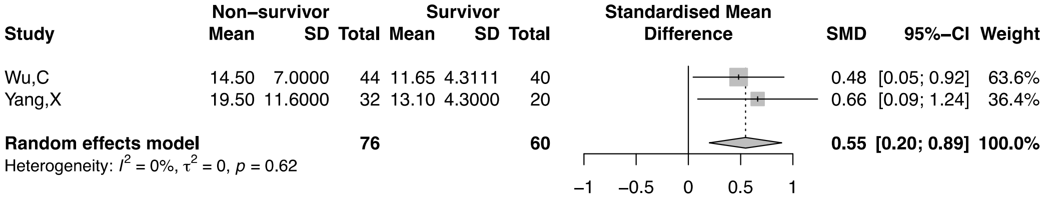

Creatinemia

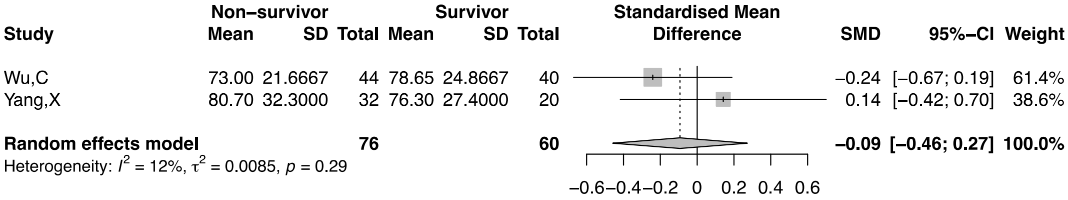

LDH

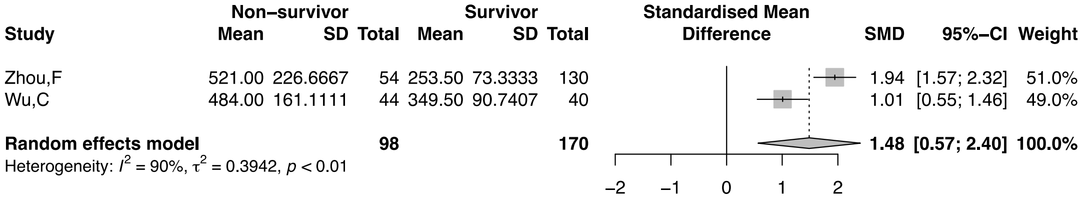

Ferritin

Study	Non–survivor Mean	SD	Total	Survivor Mean	SD	Total	SMD	95%–CI	Weight
Zhou,F	1435.30	941.5556	46	503.20	487.0370	82	1.35	[0.95; 1.75]	50.3%
Wu,C	1096.21	1067.1037	44	853.00	1213.5111	40	0.21	[−0.22; 0.64]	49.7%
**Random effects model**			**90**			**122**	**0.79**	**[−0.33; 1.90]**	**100.0%**

Heterogeneity: $I^2 = 93\%$, $\tau^2 = 0.6065$, $p < 0.01$

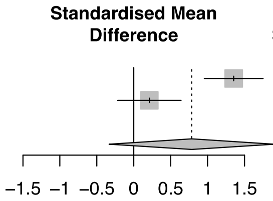

II. Forest plot of studies reporting proportion of participants with each biomarker's abnormalities

Odds of blood routine abnormalities

Lymphopenia

Study	Severe Events	Severe Total	Non–severe Events	Non–severe Total	RR	95%–CI	Weight
Wan,S	32	40	36	95	2.11	[1.56; 2.85]	12.0%
Wang,Z	11	14	17	53	2.45	[1.52; 3.95]	9.7%
Xu,Y	12	41	2	9	1.32	[0.35; 4.89]	3.1%
Li,Y	8	9	14	16	1.02	[0.76; 1.37]	12.1%
Chen,G	8	11	1	10	7.27	[1.09; 48.35]	1.7%
Li,K	22	25	22	58	2.32	[1.62; 3.32]	11.3%
Huang,C	11	13	15	28	1.58	[1.04; 2.39]	10.5%
Zheng,F	13	30	29	131	1.96	[1.16; 3.29]	9.1%
Qiu,H	9	19	2	17	4.03	[1.01; 16.09]	2.8%
Guan,W	147	153	584	726	1.19	[1.14; 1.25]	14.3%
Zhang,J	46	56	58	82	1.16	[0.96; 1.40]	13.4%
**Random effects model**		**411**		**1225**	**1.66**	**[1.26; 2.20]**	**100.0%**

Heterogeneity: $I^2 = 87\%$, $\tau^2 = 0.1234$, $p < 0.01$

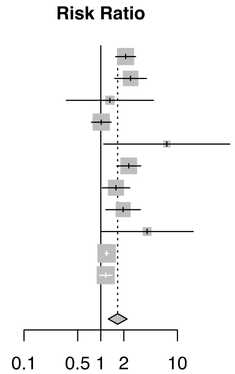

Leucopenia

Study	Severe Events	Severe Total	Non–severe Events	Non–severe Total	RR	95%–CI	Weight
Wan,S	4	40	24	95	0.40	[0.15; 1.07]	12.0%
Wang,Z	3	14	3	53	3.79	[0.85; 16.76]	9.2%
Li,Y	1	9	5	16	0.36	[0.05; 2.59]	6.9%
Chen,G	0	11	3	10	0.03	[0.00; 15.04]	1.1%
Li,K	4	25	6	58	1.55	[0.48; 5.01]	10.9%
Huang,C	1	13	9	27	0.23	[0.03; 1.63]	7.0%
Zheng,F	9	30	66	131	0.60	[0.34; 1.06]	14.4%
Qiu,H	5	19	2	17	2.24	[0.50; 10.06]	9.1%
Guan,W	102	167	228	811	2.17	[1.84; 2.56]	15.9%
Zhang,J	9	56	18	82	0.73	[0.35; 1.51]	13.6%
**Random effects model**		**384**		**1300**	**0.93**	**[0.46; 1.86]**	**100.0%**

Heterogeneity: $I^2 = 83\%$, $\tau^2 = 0.7698$, $p < 0.01$

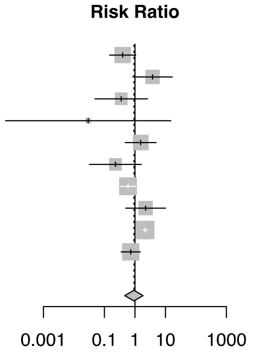

Thrombocytopenia

Study	Severe Events	Severe Total	Non–severe Events	Non–severe Total	RR	95%–CI	Weight
Wan,S	12	40	11	95	2.59	[1.25; 5.38]	5.2%
Chen,G	0	11	1	10	0.08	[0.00; 50.65]	0.1%
Huang,C	1	13	1	27	2.08	[0.14; 30.65]	0.4%
Zheng,F	3	30	8	131	1.64	[0.46; 5.81]	1.7%
Guan,W	90	156	225	713	1.83	[1.54; 2.17]	92.7%
**Random effects model**		**250**		**976**	**1.86**	**[1.59; 2.17]**	**100.0%**

Heterogeneity: $I^2 = 0\%$, $\tau^2 = 0$, $p = 0.78$

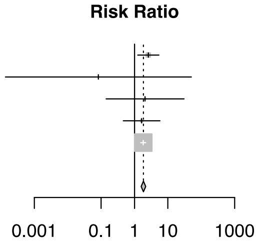

Thrombocytosis

Study	Severe Events	Severe Total	Non–severe Events	Non–severe Total	Risk Ratio	RR	95%–CI	Weight
Wan,S	28	40	84	95		0.79	[0.64; 0.98]	46.0%
Huang,C	12	13	26	27		0.96	[0.81; 1.14]	54.0%
**Random effects model**		**53**		**122**		**0.88**	**[0.26; 2.95]**	**100.0%**

Heterogeneity: $I^2 = 63\%$, $\tau^2 = 0.0169$, $p = 0.10$

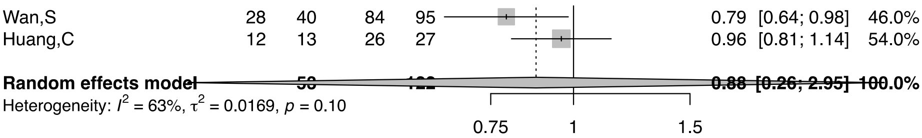

Odds of inflammatory biomarkers abnormalities

Eleveted procalcitonin

Study	Severe Events	Severe Total	Non–severe Events	Non–severe Total	Risk Ratio	RR	95%–CI	Weight
Wan,S	19	40	6	95		7.52	[3.25; 17.42]	9.5%
Wang,Z	0	12	4	50		0.10	[0.00; 51.87]	0.2%
Chen,G	4	10	1	8		3.20	[0.44; 23.28]	2.1%
Li,K	21	25	23	58		2.12	[1.48; 3.04]	24.1%
Huang,C	6	12	6	27		2.25	[0.91; 5.56]	8.5%
Qiu,H	5	19	1	17		4.47	[0.58; 34.57]	2.0%
Guan,W	16	117	19	516		3.71	[1.97; 7.00]	13.9%
Zhang,J	25	50	16	68		2.12	[1.28; 3.54]	17.9%
Wang,D	27	36	22	102		3.48	[2.30; 5.27]	21.7%
**Random effects model**		**321**		**941**		**2.94**	**[2.09; 4.15]**	**100.0%**

Heterogeneity: $I^2 = 35\%$, $\tau^2 = 0.0629$, $p = 0.14$

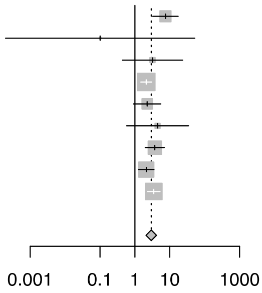

Eleveted CRP

Study	Severe Events	Severe Total	Non–severe Events	Non–severe Total	Risk Ratio	RR	95%–CI	Weight
Wang,Z	11	14	31	51		1.29	[0.91; 1.84]	12.3%
Xu,Y	24	41	2	9		2.63	[0.76; 9.19]	1.9%
Li,Y	7	9	9	16		1.38	[0.79; 2.41]	7.2%
Li,K	23	25	27	58		1.98	[1.47; 2.67]	14.1%
Zheng,F	30	30	91	131		1.44	[1.28; 1.61]	21.2%
Qiu,H	1	19	0	17		9.85	[0.02; 6172.53]	0.1%
Guan,W	110	135	371	658		1.45	[1.30; 1.60]	21.5%
Zhang,J	53	55	72	81		1.08	[0.99; 1.19]	21.8%
**Random effects model**		**328**		**1021**		**1.41**	**[1.17; 1.70]**	**100.0%**

Heterogeneity: $I^2 = 81\%$, $\tau^2 = 0.0361$, $p < 0.01$

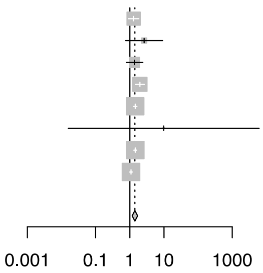

Eleveted ESR

Study	Severe Events	Severe Total	Non–severe Events	Non–severe Total	Risk Ratio	RR	95%–CI	Weight
Wang,Z	12	13	18	45		2.31	[1.56; 3.41]	90.1%
Li,Y	5	9	3	16		2.96	[0.91; 9.60]	9.9%
Qiu,H	0	19	0	17				0.0%
**Random effects model**		**41**		**78**		**2.37**	**[0.91; 6.12]**	**100.0%**

Heterogeneity: $I^2 = 0\%$, $\tau^2 = 0$, $p = 0.67$

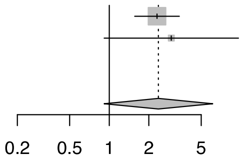

Odds of blood clothing abnormalities

Eleveted D-dimer

Study	Severe Events	Severe Total	Non–severe Events	Non–severe Total	Risk Ratio	RR	95%–CI	Weight
Li,Y	5	9	6	16		1.48	[0.63; 3.51]	8.1%
Guan,W	65	109	195	451		1.38	[1.14; 1.66]	73.5%
Zhang,J	23	38	12	43		2.17	[1.26; 3.74]	18.4%
**Random effects model**		**156**		**510**		**1.51**	**[0.89; 2.56]**	**100.0%**

Heterogeneity: $I^2 = 18\%$, $\tau^2 = 0.0137$, $p = 0.30$

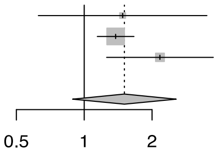

Odss of biochemical abnormalities

Eleveted LDH

Study	Severe Events	Severe Total	Non–severe Events	Non–severe Total	RR	95%–CI	Weight
Wan,S	30	40	28	95	2.54	[1.78; 3.64]	18.0%
Wang,Z	10	12	15	49	2.72	[1.66; 4.45]	14.0%
Li,Y	6	9	8	16	1.33	[0.68; 2.61]	9.9%
Chen,G	10	11	1	10	9.09	[1.40; 58.91]	1.9%
Huang,C	12	13	17	27	1.47	[1.05; 2.04]	19.0%
Zheng,F	15	30	23	131	2.85	[1.70; 4.77]	13.4%
Guan,W	72	124	205	571	1.62	[1.34; 1.95]	23.7%
**Random effects model**		**239**		**899**	**2.03**	**[1.42; 2.90]**	**100.0%**

Heterogeneity: $I^2 = 63\%$, $\tau^2 = 0.0694$, $p = 0.01$

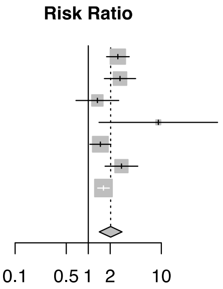

Eleveted ALAT

Study	Severe Events	Severe Total	Non–severe Events	Non–severe Total	RR	95%–CI	Weight
Cai,Qi	70	85	117	233	1.64	[1.40; 1.93]	74.7%
Wang,Z	6	14	17	55	1.39	[0.67; 2.86]	3.7%
Zheng,F	5	30	8	131	2.73	[0.96; 7.76]	1.8%
Qiu,H	0	19	2	17	0.04	[0.00; 23.45]	0.0%
Guan,W	38	135	120	606	1.42	[1.04; 1.94]	19.8%
**Random effects model**		**283**		**1042**	**1.60**	**[1.34; 1.90]**	**100.0%**

Heterogeneity: $I^2 = 0\%$, $\tau^2 = 0$, $p = 0.51$

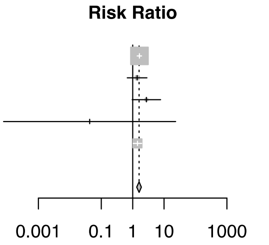

Eleveted ASAT

Study	Severe Events	Severe Total	Non–severe Events	Non–severe Total	RR	95%–CI	Weight
Cai,Qi	64	85	86	233	2.04	[1.66; 2.51]	38.7%
Wan,S	15	40	15	95	2.38	[1.29; 4.38]	9.4%
Wang,Z	7	14	12	55	2.29	[1.11; 4.73]	7.0%
Chen,G	5	11	0	10	46.41	[0.09; 22868.79]	0.1%
Huang,C	8	13	7	28	2.46	[1.14; 5.33]	6.3%
Zheng,F	12	30	10	131	5.24	[2.50; 10.98]	6.8%
Qiu,H	1	19	2	17	0.45	[0.04; 4.50]	0.8%
Guan,W	56	142	112	615	2.17	[1.66; 2.82]	31.0%
**Random effects model**		**354**		**1184**	**2.27**	**[1.76; 2.94]**	**100.0%**

Heterogeneity: $I^2 = 21\%$, $\tau^2 = 0.0167$, $p = 0.26$

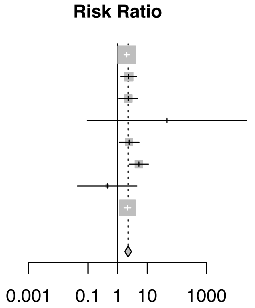

Eleveted CK

Study	Severe Events	Severe Total	Non–severe Events	Non–severe Total	RR	95%–CI	Weight
Wan,S	7	40	3	95	5.54	[1.51; 20.36]	13.5%
Huang,C	6	13	7	27	1.78	[0.75; 4.24]	22.1%
Zheng,F	9	30	8	131	4.91	[2.07; 11.68]	22.1%
Qiu,H	1	19	0	17	9.85	[0.02; 6172.53]	0.8%
Guan,W	23	121	67	536	1.52	[0.99; 2.34]	35.7%
Zhang,J	3	25	1	35	4.20	[0.46; 38.06]	5.8%
**Random effects model**		**248**		**841**	**2.61**	**[1.35; 5.05]**	**100.0%**

Heterogeneity: $I^2 = 43\%$, $\tau^2 = 0.1896$, $p = 0.12$

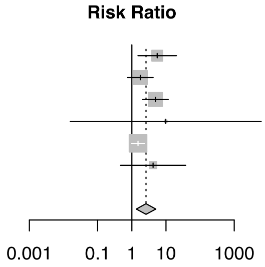

Eleveted Creatininemia

Study	Severe Events	Severe Total	Non–severe Events	Non–severe Total	RR	95%–CI	Weight
Wan,S	3	40	3	95	2.38	[0.50; 11.27]	30.6%
Zheng,F	1	30	1	131	4.37	[0.28; 67.85]	9.9%
Qiu,H	0	19	0	17			0.0%
Guan,W	6	138	6	614	4.45	[1.46; 13.59]	59.5%
**Random effects model**		**227**		**857**	**3.66**	**[1.53; 8.81]**	**100.0%**

Heterogeneity: $I^2 = 0\%$, $\tau^2 = 0$, $p = 0.81$

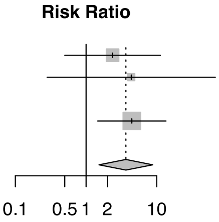

Eleveted totla bilirubin

Study	Severe Events	Severe Total	Non−severe Events	Non−severe Total	Risk Ratio	RR	95%−CI	Weight
Zheng,F	3	30	6	131		2.18	[0.58; 8.24]	12.6%
Guan,W	17	128	59	594		1.34	[0.81; 2.21]	87.4%
**Random effects model**		**158**		**725**		**1.42**	**[0.18; 11.26]**	**100.0%**

Heterogeneity: $I^2 = 0\%$, $\tau^2 = 0$, $p = 0.50$

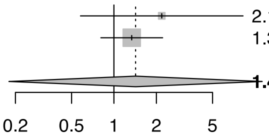

Supplement: Supplementary file 1 — Additional file 1 Supplementary Table 1. Search strategy in PubMed. Supplementary Table 2. Search strategy in EMBASE. Supplementary Figure 1. The review process. [file 40364_2020_217_MOESM1_ESM.pdf]
